# Autocrine CysLT_1_R-ERK/YAP signaling drives melanoma progression and reveals a targetable oncogenic GPCR axis

**DOI:** 10.1038/s41388-026-03919-1

**Published:** 2026-07-24

**Authors:** Emma Elizabeth Sabu Kattuman, Lakshminarayan Reddy Teegala, Venkatesh Katari, Somayeh Darzi, Srinivas Vinod Saladi, Ivana de la Serna, Charles K. Thodeti, Sailaja Paruchuri

**Affiliations:** 1 Department of Physiology and Pharmacology, The University of Toledo College of Medicine and Life Sciences, Toledo, OH USA; 2 Department of Cell and Cancer Biology, The University of Toledo College of Medicine and Life Sciences, Toledo, OH USA

**Keywords:** Melanoma, Lipid signalling, Inflammation

## Abstract

Cutaneous melanoma remains the most lethal skin cancer due to profound tumor heterogeneity and the frequent development of resistance to current therapies. Here, we identify the cysteinyl leukotriene receptor 1 (CysLT_1_R) as a previously unrecognized driver of melanoma progression. Analysis of bulk RNA-sequencing datasets from The Cancer Genome Atlas (TCGA) revealed significantly elevated CysLT_1_R transcript in metastatic tumors compared to primary tumors. Functional studies in murine and human melanoma cells demonstrated that leukotriene D_4_ (LTD_4_)-mediated activation of CysLT_1_R promotes melanoma cell proliferation and invasion through the parallel engagement of YAP and ERK signaling pathways. Notably, melanoma cells express LTC_4_ synthase and secrete cysteinyl leukotrienes, establishing a constitutive autocrine signaling loop that sustains CysLT_1_R activity independently of the host niche. Genetic ablation or pharmacological inhibition of CysLT_1_R with MK571 significantly attenuated tumor growth in vivo and was associated with inhibition of the YAP-LOXL-2 signaling axis. In addition, studies using *Cysltr1*^–/–^ mice reveal that host-derived CysLT_1_R signaling within the tumor microenvironment also contributes to melanoma progression. Together, these findings uncover a previously unrecognized pro-tumorigenic CysLT_1_R-ERK/YAP,LOXL-2 signaling circuit that promotes cutaneous melanoma progression and highlight CysLT_1_R as a potential therapeutic target for melanoma.

## Introduction

Cutaneous melanoma, characterized by high metastatic potential and profound heterogeneity, remains the leading cause of skin cancer-related mortality worldwide [1, 2]. Despite the clinical success of targeted immunotherapies, 5-year survival for stage IV metastatic disease remains approximately 31% [3], primarily due to the emergence of therapy resistance, demanding novel therapeutic targets. Chronic inflammation is the fundamental driver of this malignant progression [4, 5]. Specifically, cysteinyl leukotrienes (cys-LTs), comprising leukotriene (LT) C_4_ (LTC_4_), LTD_4_, and LTE_4_, have emerged as potent bioactive lipids within the tumor microenvironment (TME) produced by tissue-infiltrating immune cells [6]. Cys-LTs are synthesized from arachidonic acid via 5-lipoxygenase (5-LO)/ 5-LO-activating protein (FLAP)/ LTC_4_ synthase (LTC_4_S) pathway and exert their inflammatory effects via three G protein-coupled receptors (GPCRs), cysteinyl leukotriene receptor 1 (CysLT_1_R), cysteinyl leukotriene receptor 2 (CysLT_2_R) [7], and recently identified cysteinyl leukotriene receptor 3 (CysLT_3_R) [8]. CysLT_1_R has high affinity for LTD_4_ compared to LTC_4_ and LTE_4_ [9], CysLT_2_R has equal affinities for both LTC_4_ and LTD_4_ followed by LTE_4_ [10], and CysLT_3_R has high affinity for LTE_4_ [8]. While CysLT_1_R has been implicated in the progression of various malignancies and correlates with poor survival in breast [11] and uveal melanoma [12, 13], its functional contribution to cutaneous melanoma remains poorly understood. We and others have previously demonstrated that CysLT_1_R is a critical regulator of cellular processes including proliferation and migration in normal [14–19], and tumor cells [20–22]. However, the molecular mechanisms through which it coordinates melanoma growth and its potential as a therapeutic target have not been elucidated. In this study, employing both mouse and human melanoma cells, we demonstrate that CysLT_1_R/Gαq signaling is a critical driver of melanoma cell proliferation, migration and invasion via parallel ERK-YAP/LOXL-2 pathways. Notably, we uncovered an autocrine loop where melanoma cells synthesized cys-LTs to maintain constitutive CysLT_1_R activity. In vivo, by integrating CysLT_1_R global knockout mice (*Cysltr1*^*–/–*^) alongside the CysLT_1_R antagonist MK571, we reveal that CysLT_1_R drives tumor progression through both tumor-intrinsic and host-extrinsic mechanisms. Our findings identify a novel CysLT_1_R-YAP/LOXL-2 signaling axis promoting melanoma tumors, positioning CysLT_1_R as a vital target for drug repurposing.

## Materials and methods

### Cells

Murine B16F10 and YUMM1.7 melanoma cell lines, along with human melanoma cell line, WM266-4, were kindly gifted by Dr. Ivana de la Serna, a Professor in the Department of Cell and Cancer Biology at the University of Toledo, OH, who purchased the cell lines from ATCC (Manassas, VA, USA). All cell lines were cultured in Dulbecco’s Modified Eagle’s Medium (DMEM) (Invitrogen, Carlsbad, CA, USA), supplemented with 10% fetal bovine serum (FBS) (Phoenix Scientific, San Marcos, CA, USA), 100 units/ml penicillin-streptomycin, 2 mM L-glutamine (Invitrogen, Carlsbad, CA, USA), and maintained at 37 °C in a humidified 5% CO_2_ environment. All cells were used at low passages, and comparable passages were used in different experiments.

### Reagents

The reagents and chemicals were purchased commercially: LTD_4_, CysLT_1_R antagonist (MK571), and cys-LT ELISA kit from Cayman Chemicals, Ann Arbor, MI, USA, phospho- and total-antibodies of ERK, P38, AKT, YAP, and LOXL-2 from Cell signaling technology, Danvers, MA, USA, GAPDH antibody from Fitzgerald, Acton, MA, USA, all secondary antibodies from Jackson Immuno Research, West Grove, PA, USA, transcriptor first strand cDNA synthesis kit and light cycler 480 SYBR Green I Master Mix from Roche, Indianapolis, IN, USA, Inhibitors for ERK/MEK pathway (PD98059), PI3K/AKT pathway (LY294002), and P38/MAPK pathway (BIRB0796) from Tocris Bioscience, Minneapolis, MN, USA, CysLT_1_RsiRNA, NSsiRNA, and all reagents for receptor knockdown from Dharmacon^TM^, Lafayette, CO, USA, all reagents for western blot analysis from Bio-Rad, Hercules, CA, USA, primers for qPCR from Integrated DNA Technologies, Coralville, IO, USA, Fura-2AM from Thermo Fisher Scientific^TM^, Waltham, MA, USA, WoundMaker^TM^ from Essen BioScience, Ann Arbor, MI, USA, 8.0 µm trans-well inserts from Corning, NY, BrDU proliferation assay kit from EMD Millipore Corporation, La Jolla, CA, USA.

### Animals

All in vivo experiments were performed on 6-8-week-old WT and *Cysltr1*^*–/–*^ mice. WT mice were purchased from The Jackson Laboratory, and *Cysltr1*^*–/–*^ mice on a C57BL/6 background were kindly provided by Dr. K. Frank Austen’s lab, Brigham and Women’s Hospital, Boston [23]. Both strains were maintained at the Department of Laboratory Animals and Research (DLAR) at the University of Toledo. All animal experiments were done in accordance with standard guidelines as approved by the Institutional Animal Care and Use Committee of the University of Toledo. Both male and female mice were used for the experiments.

### Ethics statement

All animal experiments were approved by the Institutional Animal Care and Use Committee of the University of Toledo (Protocol No: 400209) and performed in accordance with institutional guidelines and the NIG Guide for the Care and Use of Laboratory Animals. Publicly available TCGA datasets were analyzed in accordance with their data access policies and required no additional ethical approval. We used BioRender to create some of the illustrations, and we have obtained permission from them to publish them in a journal.

### TCGA database

Raw bulk RNA sequence data of 472 melanoma patients were extracted from The Cancer Genome Atlas (TCGA) database. CysLT_1_R gene expression in primary and metastatic tumors was segregated and represented as Z scores based on the stage of melanoma progression at the time of biopsy collection.

### Calcium flux assay

YUMM1.7, B16F10, and WM266-4 cells were cultured on MatTek glass-bottomed dishes and pre-treated with Fluo-4 for 30 min. After incubation, cells were briefly washed thrice using calcium buffer (in mM: 136 NaCl, 4.7 KCl, 1.2 MgSO_4_, 1.1 CaCl_2_, 1.2 KH_2_PO_4_, 5 NaHCO_3_, 5.5 glucose, and 20 HEPES, pH 7.4). Cells were then imaged under a Leica confocal microscope (SP5), and calcium flux was observed in response to LTD_4_ stimulation with or without CysLT_1_R antagonist (MK571) pre-treatment for 10 min. Images were acquired every 1 s for 2 mins and analyzed using Leica software.

### Cell activation and treatments

B16F10 and YUMM1.7 cells were pre-treated with antagonists of CysLT_1_R (MK571, 1 µM) and ERK (PD98059, 50 µM) for 30 min followed by stimulation with LTD_4_ (100 nM or 500 nM) for the indicated time to analyze the expression of p-ERK, p-P38, p-AKT, p-YAP, and their totals along with LOXL-2 and GAPDH.

### CysLT_1_R knockdown using siRNA transfection

For all experiments involving CysLT_1_R knockdown, YUMM1.7 cells were transfected with siGENOME SMART pool (a mix of 4 pre-made siRNAs from Dharmacon^TM^, Lafayette, CO, to block CysLT_1_R (20 nmol/L), or they were transfected with non-specific siRNA (negative control). Transfection was performed with siLentFect transfection reagent (Bio-Rad) as per the manufacturer’s instructions.

### Cell lysates and western blotting

Following stimulations or gene knockdown, melanoma cells (2.5 × 10^5^ cells per well) were lysed with lysis buffer (BD Bioscience, San Jose, CA, USA) supplemented with Protease and Phosphatase Inhibitor Cocktails (Thermo Fisher Scientific, Waltham, MA). Immunoblotting was performed as described previously [15]. Briefly, lysates were subjected to 4-12% SDS-PAGE and transferred to a PVDF membrane. Membranes were incubated with respective primary phospho- and total antibodies diluted in 1x TBS, 5% dry milk, 0.1% Tween-20 (1:1000) overnight at 4°C on a shaker, and then with secondary antibody (peroxidase-conjugated anti-rabbit or anti-mouse) (1:5000). Western blot was incubated with ECL and the bands were visualized using an imager (Azure 500, Dublin, CA) and quantified using Image J. Densitometric analysis was performed by normalizing the respective bands to the loading control.

### Real-time quantitative PCR

Gene expressions in all three murine and human melanoma cell lines with or without genetic knockdown of CysLT_1_R were determined by qPCR performed using LightCycler 480 (Roche). Total RNA was isolated from the cells after respective treatments and cDNA synthesis was performed as preciously described [24]. qPCR was performed using the primers mentioned in supplementary table 1. The expression levels of respective genes relative to GAPDH were analyzed and the 2^-ΔCp^ values were calculated and expressed as a relative expression or fold change compared to the control.

### Cell proliferation

To determine cell proliferation, 10^4^ cells per well of melanoma cells were stimulated with LTD_4_ (10 nM or 100 nM) with or without MK571 (1 µM), PD98059 (50 µM), LY294002 (50 µM), BIRB0796 (1 µM), and verteporfin (1 µM) for 72 h. Proliferation was measured after the incubation period using the BrdU assay, according to the manufacturer’s protocol. BrdU label was added 24 h before the termination of the incubation.

### 2D cell migration

Melanoma cell migration was determined using the scratch wound assay. 10^4^ cells (B16F10 and WM266-4) per well were seeded in a 96-well plate. When the cells reached almost 100% confluency, a scratch was made in the center of each well using the WoundMaker^TM^. Cells were stimulated with LTD_4_ (10 nM or 500 nM), and each treatment had a minimum of 6 replicates. Plate was then transferred to IncuCyte® S3 Live-Cell Analysis System to capture the closure of the scratch wound by the cells over a period of 24-48 h. Results were analyzed using Olyvia software.

### 2D cell invasion

Melanoma cell invasion was determined using a transwell assay. 3 × 10^5^ cells in 200 µL serum-free media were seeded on Matrigel^®^ pre-coated trans-well inserts of 8.0 µm pore size placed in a 24-well plate. The bottom chamber was supplied with 750 μL of 10% FBS media acting as a chemoattractant for invading cells. Both top and bottom chamber media received stimulations with or without LTD_4_ (100 nM), MK571 (1 µM), PD98059 (50 µM), and verteporfin (1 µM). The plate was incubated for 24 h in a 5% CO_2_ humidified environment at 37°C. To end the experiment, cells on the bottom of the trans-well insert were fixed with 4% PFA for 15 min followed by staining with 0.1% crystal violet solution for 30 min. Cells on the top of the membrane were physically removed using a cotton swab. Membranes were then mounted onto glass slides, and five sites were randomly imaged under a light microscope. For quantification, membranes were de-stained with 100% methanol for 15 min. Intensity of crystal violet in methanol solution determined by the absorbance reading (590 nm) was considered to be directly proportional to the number of invaded cells.

### Cys-LT ELISA

Concentration of cys-LTs in the supernatant of YUMM1.7, B16F10, and WM266-4 cells treated in the presence or absence of TNF-α (10 ng/mL) for a period of 12 h for YUMM1.7 and WM266-4 cells, and 24 h for B16F10 cells, was assayed using cys-LT ELISA kit from Cayman chemicals, Ann Arbor, MI, according to the manufacturer’s protocol.

### Ectopic tumor model

For in vivo ectopic tumor induction, B16F10 and YUMM1.7 cells (10^6^ per flank of each mouse) were subcutaneously injected under the flank of 6-8-week-old WT and *Cysltr1*^*–/–*^ mice with 27-G needles. Tumor volume was measured using a digital caliper at 7, 14, 18 and 21 days after tumor cell injections. Tumor volumes were determined using the formula 4/3*π*(l/2)*(w/2)^2^, where “l” is the longest diameter and “w” is the perpendicular length of the tumor. On day 21, mice were euthanized, and primary tumors were harvested.

### Orthotopic tumor model

To mimic the natural tumor microenvironment, we performed intradermal implantation of melanoma cells in mice. 0.1×10^6^ YUMM1.7 cells in 50 μL of HBSS media were intradermally injected into each flank of WT and *Cysltr1*^*−/−*^ mice of either sex with 30-G needles. Tumor volumes were measured as above. Mice were euthanized on day 21, and tumors were harvested. RNA and protein were isolated from the tumors, and qPCR and western blot were performed to analyze the expression of YAP, LOXL-2, and ECM markers.

### Pharmacological inhibition of CysLT_1_R in vivo

After ectopically or orthotopically inducing tumors in WT mice using YUMM1.7 and B16F10 cells, when tumors were palpable on the seventh day (around 100mm^3^), WT mice were divided into 2 cohorts. First cohort of mice received 150 µL of saline by intraperitoneal (i.p) injection, whereas the second cohort received MK 571 (3 mg/kg of body weight in 150 µL of saline) by intraperitoneal (i.p) injection. Saline and MK571 were administered every three days till day 21, when mice were euthanized, and primary tumors were harvested. Tumor volumes were measured as previously described. Upon euthanasia, RNA and protein were isolated from tumors and analyzed for the same markers as above.

### Statistical analysis

All results are representative of at least three independent experiments performed, and data were expressed as mean ± SD except where otherwise indicated. D’Agostino & Pearson test was performed to test if the sample distribution is normal, and significance was determined using Student t-test for two groups and one-way analysis of variance (ANOVA) for more than two groups, followed by Tukey’s multiple comparisons test for post hoc analysis using GraphPad Prism 8.

## Results

### CysLT_1_R expression is upregulated with cutaneous melanoma progression

**Fig. 1:**
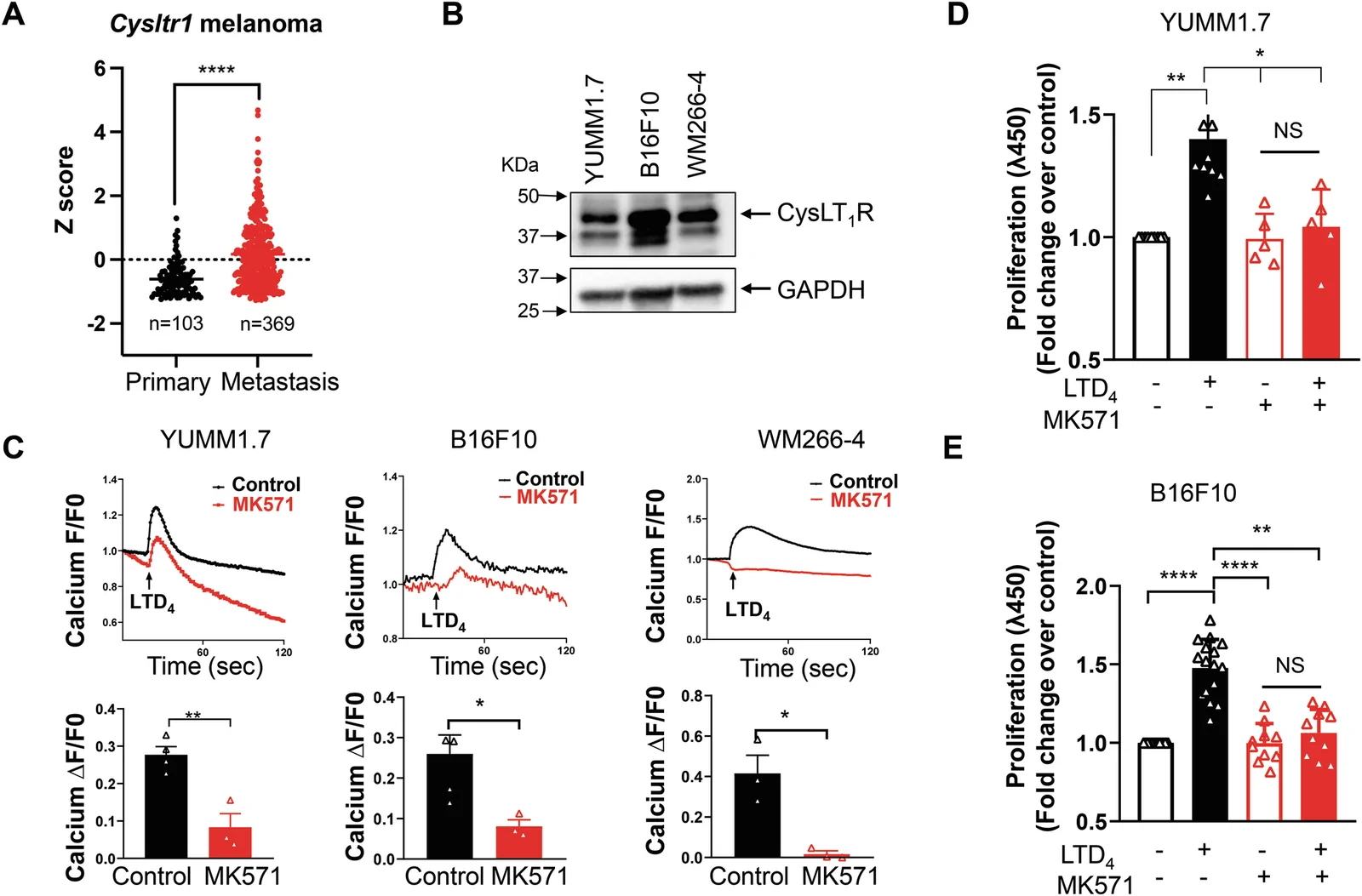
CysLT_1_R is upregulated with melanoma progression, and LTD_4_ fluxes calcium and promotes melanoma cell proliferation via CysLT_1_R. **A** Z-scores of CysLT_1_R bulk RNA-seq data of cutaneous melanoma patient tumors at primary and metastasis stage extracted from TCGA database (*n* = 472) [25]. **B** Western blot analysis of CysLT_1_R protein in two murine melanoma cell lines (YUMM1.7 and B16F10) and one human melanoma cell line (WM266-4). **C** Representative traces display relative changes in cytosolic calcium in response to 500 nM LTD_4_, in Fluo-4-loaded cells pre-treated with or without CysLT_1_R antagonist, MK571 (1 μM) in YUMM1.7, B16F10 and WM266-4 cells. **D** YUMM1.7 and **E** B16F10 cells were stimulated with or without LTD_4_ and MK571 (CysLT_1_R antagonist) for 72 h followed by BrdU assay to evaluate their proliferation. All data representations are means ± SD from three independent experiments (*n* = 3). D’Agostino & Pearson test was performed to test if the sample distribution is normal, and significance was determined using ANOVA. Comparisons between the groups were determined by Tukey’s multiple comparisons test. *P < 0.05, **P < 0.01, ****P < 0.0001.

To determine the relevance of CysLT_1_R in melanoma patients, we analyzed bulk RNA-seq data of melanoma patient samples (*n* = 472) obtained from The Cancer Genome Atlas (TCGA) database [25]. We observed a significant upregulation of CysLT_1_R transcript expression in metastatic tumors compared to primary tumors, indicating a crucial role played by the receptor in melanoma progression (Fig. 1A).

### Murine and human melanoma cells express functional CysLT_1_R

To elucidate the role of CysLT_1_R in cutaneous melanoma, we first determined its expression in murine (YUMM1.7 and B16F10) and human (WM266-4) melanoma cells. Both murine and human melanoma cells expressed significant levels of CysLT_1_R protein (Fig. 1B). Next, to determine if the receptors are functional, we loaded melanoma cells with Fluo-4 and assessed their ability to flux calcium in response to CysLT_1_R ligand, LTD_4_. LTD_4_ induced robust calcium flux in all melanoma cell lines, and this response was significantly inhibited by pretreatment of the cells with CysLT_1_R-specific antagonist MK571 (Fig. 1C), indicating that the calcium flux elicited by LTD_4_ is via CysLT_1_R. Of note, melanoma cells also express CysLT_2_R and CysLT_3_R transcripts at levels comparable to those of CysLT_1_R (data not shown); however, the present study focuses specifically on defining the contribution of CysLT_1_R.

### LTD_4_ promotes melanoma cell proliferation via CysLT_1_R

To gain functional insights on CysLT_1_R signaling in cell proliferation, we measured proliferation in melanoma cells stimulated with LTD_4_ in the presence and absence of CysLT_1_R antagonist (MK571) using BrdU assay. LTD_4_ caused a moderate, but significant increase in proliferation in both murine melanoma cell lines (YUMM1.7 and B16F10) (Fig. 1D, E). This effect was mirrored in human melanoma cell line where LTD_4_ also promoted WM266-4 proliferation (Supp. Fig. 1A). Notably, LTD_4_ effect was subsequently inhibited by the addition of CysLT_1_R antagonist, MK571 (Fig. 1D, E), suggesting that LTD_4_ promotes melanoma cell proliferation via CysLT_1_R.

### LTD_4_/CysLT_1_R promotes melanoma cell proliferation, migration, and invasion via the ERK pathway

**Fig. 2:**
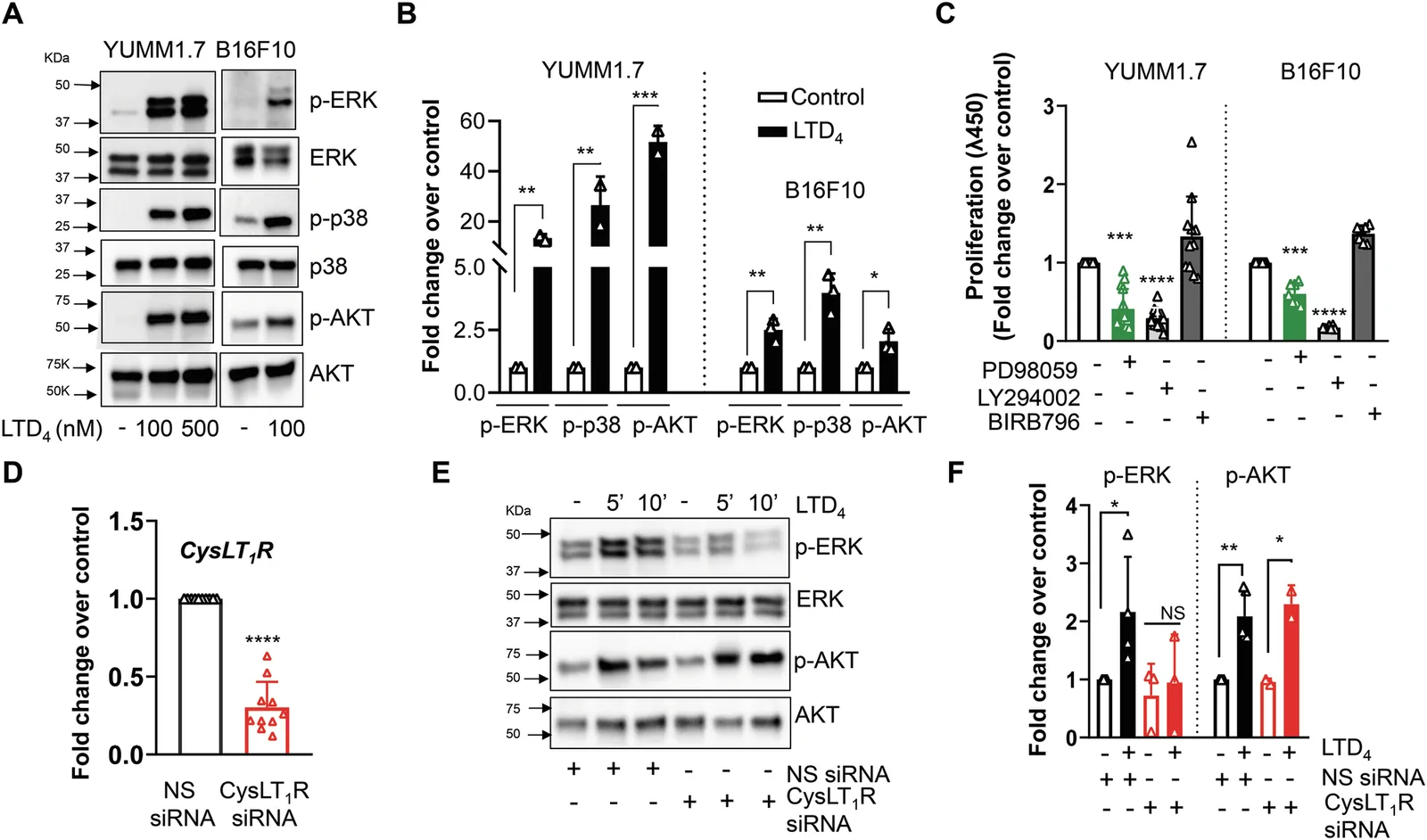
LTD_4_/CysLT_1_R promotes melanoma cell proliferation via ERK pathway. **A** YUMM1.7 and B16F10 cells were treated with indicated concentrations of LTD_4_ for 5 min. Protein expression of phospho- and total-ERK, p38, and AKT was analyzed by western blotting, and **B** quantified. **C** YUMM1.7 and B16F10 cells were treated with or without PD98059 (ERK inhibitor), LY294002 (PI3K/AKT inhibitor), and BIRB796 (p38 inhibitor) for 72 h followed by BrdU assay to evaluate cell proliferation. CysLT_1_R protein was knocked down in YUMM1.7 by transfecting them with siRNA against CysLT_1_R (20 nM). Non-specific (NS) siRNA transfected YUMM1.7 were used as a control. **D** CysLT_1_R knock down efficiency was examined through qPCR. 48 h after transfection, YUMM1.7 were treated with or without LTD_4_, and **E** phosphorylated and total ERK and AKT expression were analyzed by western blotting and **F** quantified. All data representations are means ± SD from three independent experiments (*n* = 3). D’Agostino & Pearson test was performed to test if the sample distribution is normal, and significance was determined using ANOVA. Comparisons between the groups were determined by Tukey’s multiple comparisons test. *P < 0.05, **P < 0.01, ***P < 0.001, ****P < 0.0001, NS-non-significant.

To unravel the signaling downstream of LTD_4_/CysLT_1_R axis, we analyzed phosphorylation profiles of ERK, p38, and AKT, the key proliferative signaling intermediates in both YUMM1.7 and B16F10 cells in response to LTD_4_ stimulation. LTD_4_ significantly increased levels of phosphorylated ERK, p38, and AKT, suggesting their activation in both cell lines (Fig. 2A, B). To further evaluate which of these pathways are involved in melanoma cell proliferation, we performed BrdU assay on YUMM1.7 and B16F10 cell lines in the presence or absence of specific inhibitors for each of these pathways. We found that PD98059 (ERK inhibitor) and LY294002 (AKT inhibitor), but not BIRB796 (p38 inhibitor), significantly inhibited proliferation in both the cell lines (Fig. 2C), suggesting that ERK and AKT pathways drive melanoma cell proliferation. To further determine the role of CysLT_1_R in activating these pathways, we knocked down CysLT_1_R in YUMM1.7 cells using CysLT_1_R siRNA (Fig. 2D), followed by LTD_4_ stimulation and analyzed phosphorylation of ERK and AKT. Interestingly, knockdown of CysLT_1_R inhibited LTD_4_-stimulated phosphorylation of ERK but not AKT (Fig. 2E, F). We speculate that the sustained AKT phosphorylation observed following CysLT_1_R knockdown could reflect parallel signaling via CysLT_2_R. However, as the primary objective of this study is to define the specific contribution of CysLT_1_R, we did not systematically dissect the roles of the other CysLT receptors. Our results demonstrated that the LTD_4_/CysLT_1_R axis promotes cell proliferation via the ERK pathway in melanoma cells.

**Fig. 3:**
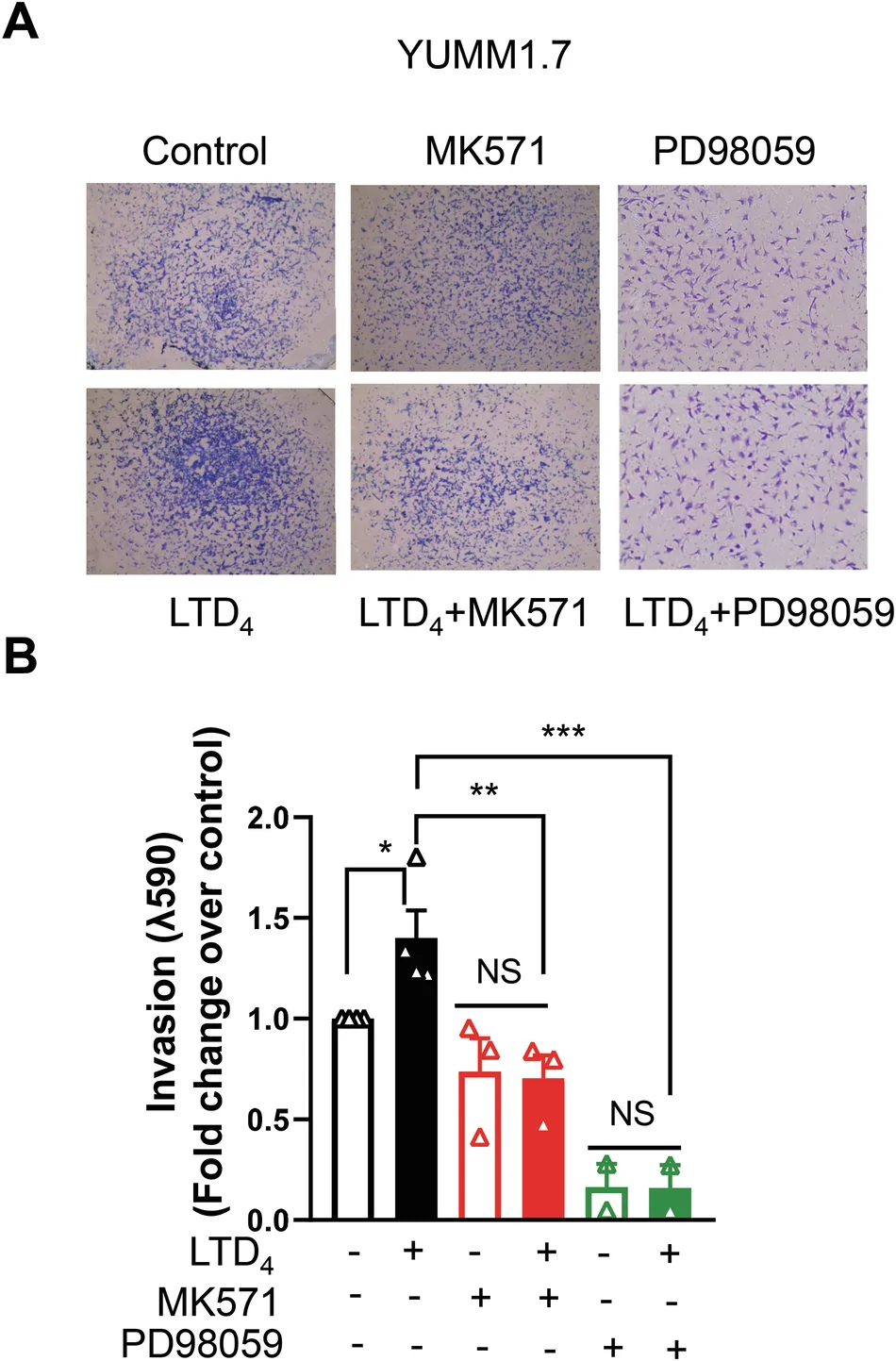
LTD_4_/CysLT_1_R promotes melanoma cell invasion via ERK pathway. 3 × 10^5^ YUMM1.7 cells in 200 µL serum-free media were seeded on Matrigel® pre-coated trans-well inserts of 8.0 µm pore size placed in a 24-well plate. The bottom chamber comprised 750 µL of 10% FBS acting as chemoattractant to stimulate the movement of the cells through the matrix. Cells were stimulated with or without LTD_4_ (100 nM) in the presence or absence of MK571 (1 µM), or PD98059 (50 µM). Cells were allowed to invade for 24 h after which membranes were fixed and **A** stained with crystal violet and **B** quantified. All data representations are means ± SD from three independent experiments (*n* = 3). D’Agostino & Pearson test was performed to test if the sample distribution is normal, and significance was determined using ANOVA. Comparisons between the groups were determined by Tukey’s multiple comparisons test. *P < 0.05, **P < 0.01, ***P < 0.001.

LTD_4_ stimulation significantly promoted migration in B16F10 and WM266-4 cells (Supplementary Fig. 1B&D) and invasion of YUMM1.7 cells compared to controls, which was inhibited by MK571 (Fig. 3A, B). Additionally, we found that the ERK inhibitor PD98059 also significantly decreased invasion of YUMM1.7 cells, further re-iterating the involvement of the ERK pathway in melanoma cell migration and invasion (Fig. 3A, B). In line with these observations, LTD_4_ also promoted invasion of WM266-4 cells (Supplementary Fig. 1C). Taking these results together, the LTD_4_/CysLT_1_R/ERK signaling pathway promoted migration and invasion of melanoma cells.

### The LTD_4_/CysLT_1_R axis also activates the mechanosensory transcriptional coactivator YAP and its downstream effector LOXL-2 to drive melanoma cell proliferation and invasion

**Fig. 4:**
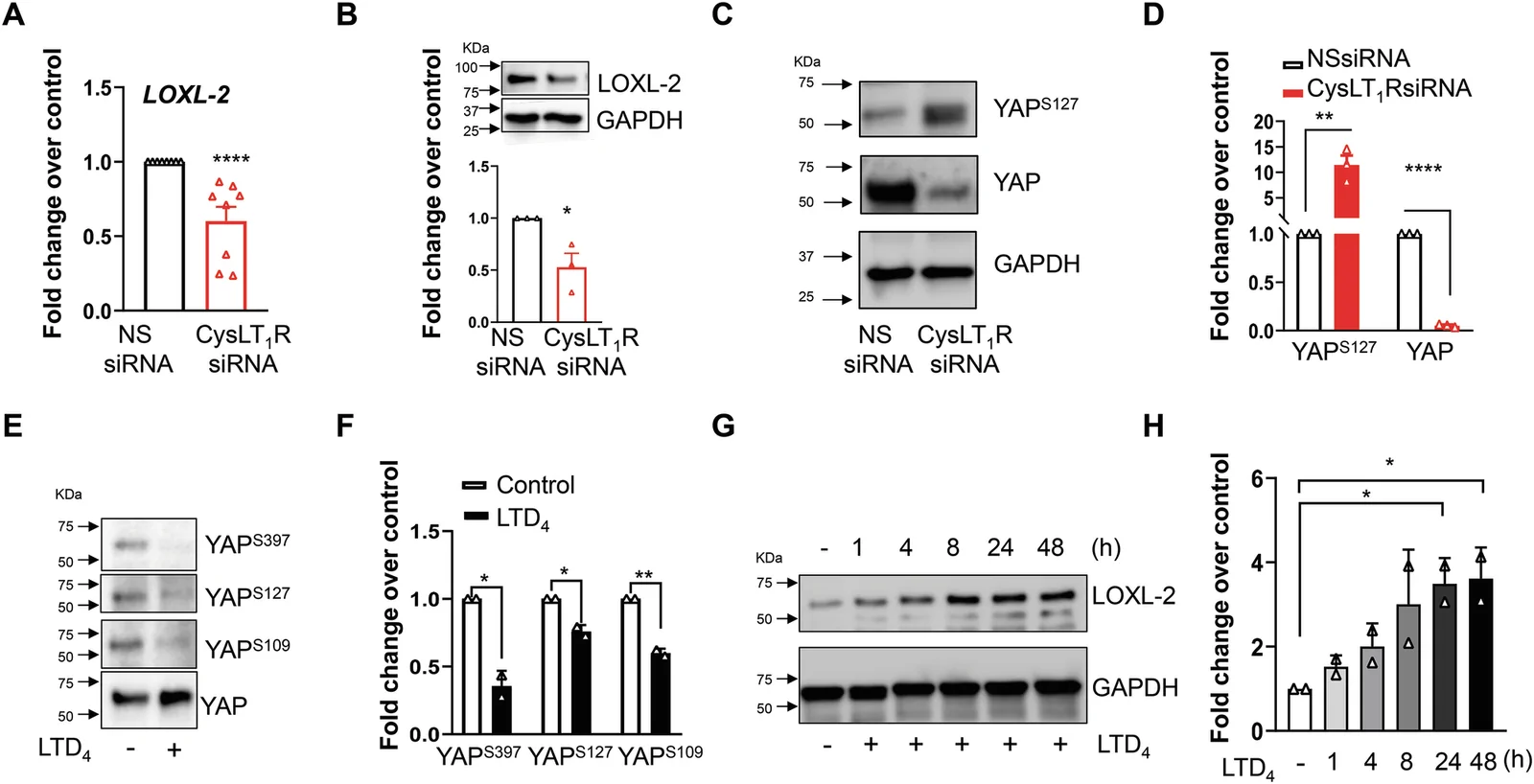
LTD_4_/CysLT_1_R activates YAP and its downstream effector, LOXL-2. Mechanosensory proteins, LOXL-2 (**A**, **B**) and YAP (**C**, **D**) expressions were determined in YUMM1.7 cells knocked down with NSsiRNA and CysLT_1_RsiRNA for 24 h at transcript (**A**) and protein (**B**–**D**) levels respectively. YUMM1.7 cells were treated with or without LTD_4_ (500 nM) for 1 h and YAP phosphorylation was determined by **E** western blot **F** quantified. **G** YUMM1.7 cells were treated with or without LTD_4_ (500 nM) in a time-dependent manner for 48 h and LOXL-2 protein expression was determined by western blot and **H** quantified. All data representations are means ± SD from three independent experiments (*n* = 3). D’Agostino & Pearson test was performed to test if the sample distribution is normal, and significance was determined using ANOVA. Comparisons between the groups were determined by Tukey’s multiple comparisons test. *P < 0.05, **P < 0.01,****P < 0.0001.

Since CysLT_1_R was important for melanoma cell migration and invasion, we investigated its involvement in regulating the extra-cellular matrix (ECM). siRNA knock down of CysLT_1_R in YUMM1.7 cells caused significant downregulation of fibronectin (FN) and vimentin expression at transcript and protein level, and several other ECM transcripts including *matrix-metalloprotease 2* (*MMP-2*), *twist1*, and *snail* (Supplementary Fig. 2A–G). Importantly, we observed that CysLT_1_R knock down led to significant downregulation of a key mechanosensory protein lysyl oxidase-like 2 (LOXL-2), implicated in cancer [26, 27] at both mRNA and protein levels (Fig. 4A, B). Further, 24 h CysLT_1_R knock down resulted in significant phosphorylation at s^127^ and inactivation of yes-associated protein (YAP), an upstream regulator of LOXL-2 (Fig. 4C, D). We also observed a simultaneous reduction in the total YAP. We speculate that the reduction in total YAP at 24 h may reflect degradation and/or turnover of phosphorylated YAP [28]. The discrepancy in signal intensity between total and phospho-YAP could be due to the differences in antibody avidity, sensitivity, and epitope accessibility between phospho-specific and total YAP antibodies. It is possible that the phospho-YAP antibody may detect low levels of phosphorylated protein more efficiently than the total YAP antibody detects total YAP protein under similar conditions.

Next, we asked if LTD_4_ can activate YAP and its downstream signaling. Stimulation of YUMM1.7 cells with LTD_4_ for 1 h led to significant reduction in YAP phosphorylation at all three phosphorylation sites (S^397^, S^127^ and S^109^), suggestive of its activation at 1 h post-LTD_4_ stimulation (Fig. 4E, F). Since LTD_4_ stimulations were done only at short time point (1 h), we did not observe reduction in the total YAP (Fig. 4E). Subsequently, LTD_4_ stimulation also steadily enhanced the expression of its downstream effector, LOXL-2 with maximum expression at 48 h post LTD_4_ treatment (Fig. 4G, H).

### YAP is vital for LTD_4_/CysLT_1_R-mediated proliferation and invasion

**Fig. 5:**
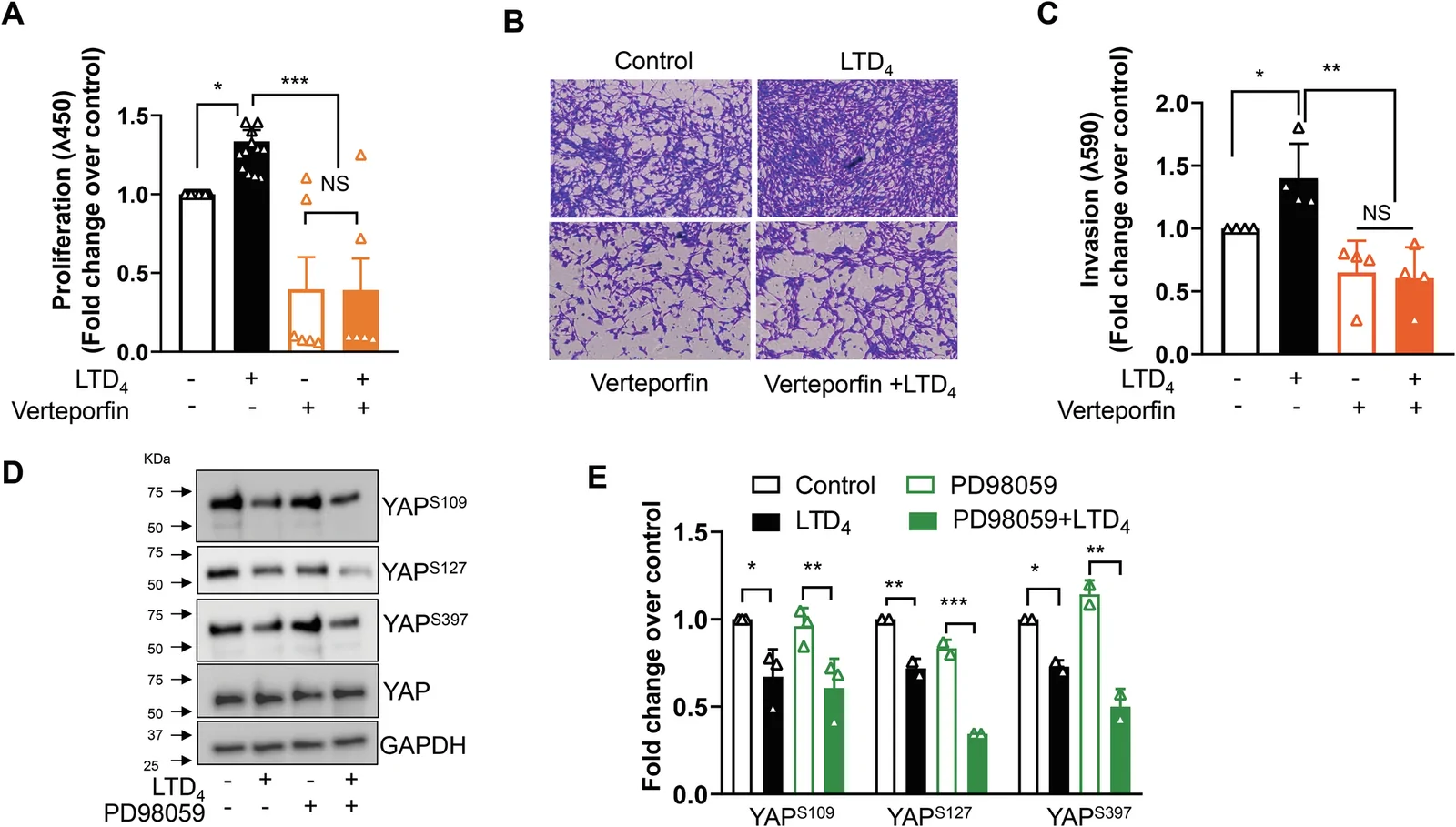
LTD_4_/CysLT_1_R promotes melanoma cell proliferation and invasion via YAP pathway which is independent of ERK signaling. **A** YUMM1.7 cells were treated with or without verteporfin (1 µM) (YAP inhibitor) and stimulated with LTD_4_ for 72 h, followed by BrdU assay to evaluate cell proliferation. 3 × 10^5^ YUMM1.7 cells in 200 µL serum-free media were seeded on Matrigel® pre-coated trans-well inserts of 8.0 µm pore size placed in a 24-well plate. The bottom chamber was supplemented with 750 µL of 10% FBS media to stimulate the movement of the cells through the matrix. Cells were treated with verteporfin (1 µM) and stimulated with or without LTD_4_ (100 nM) for 24 h, after which membranes were fixed and **B** stained with crystal violet and **C** quantified. YUMM1.7 cells were pre-treated with or without PD98059 for 30 min followed by LTD_4_ (500 nM) stimulation for 1 h. **D** YAP phosphorylation was assessed by western blot and **E** quantified. All data representations are means ± SD from three independent experiments (*n* = 3). D’Agostino & Pearson test was performed to test if the sample distribution is normal, and significance was determined using ANOVA. Comparisons between the groups were determined by Tukey’s multiple comparisons test. *P < 0.05, **P < 0.01, ***P < 0.001.

Next, we explored the role of YAP in CysLT_1_R-mediated melanoma cell proliferation and invasion using the BrdU assay and trans-well invasion assay respectively. We found that treatment of YUMM1.7 with YAP specific inhibitor, verteporfin, significantly decreased LTD_4_-induced proliferation of the cells compared to controls (Fig. 5A). Further, verteporfin pre-treatment significantly inhibited LTD_4_-induced invasion of YUMM1.7 cells (Fig. 5B, C) indicating the significance of the CysLT_1_R/YAP axis in melanoma cell invasion. Our results uncover a previously unidentified CysLT_1_R-YAP axis in regulating melanoma.

### LTD_4_/CysLT_1_R enhances melanoma cell proliferation and invasion via ERK and YAP signaling pathways in an independent manner

To determine if LTD_4_ activated ERK upstream of YAP, we pre-treated YUMM1.7 cells with ERK inhibitor, PD98059 for 30 min, followed by LTD_4_ stimulation for 1 h. LTD_4_ stimulation significantly activated YAP as determined by reduction in its phosphorylation in consensus with our earlier observation. However, this activation was unaltered in the presence of PD98059 (Fig. 5D, E) suggesting that the LTD_4_/CysLT_1_R axis promotes melanoma cell proliferation and invasion via multiple pathways by independent mechanisms.

### Autocrine cys-LT signaling in melanoma

**Fig. 6:**
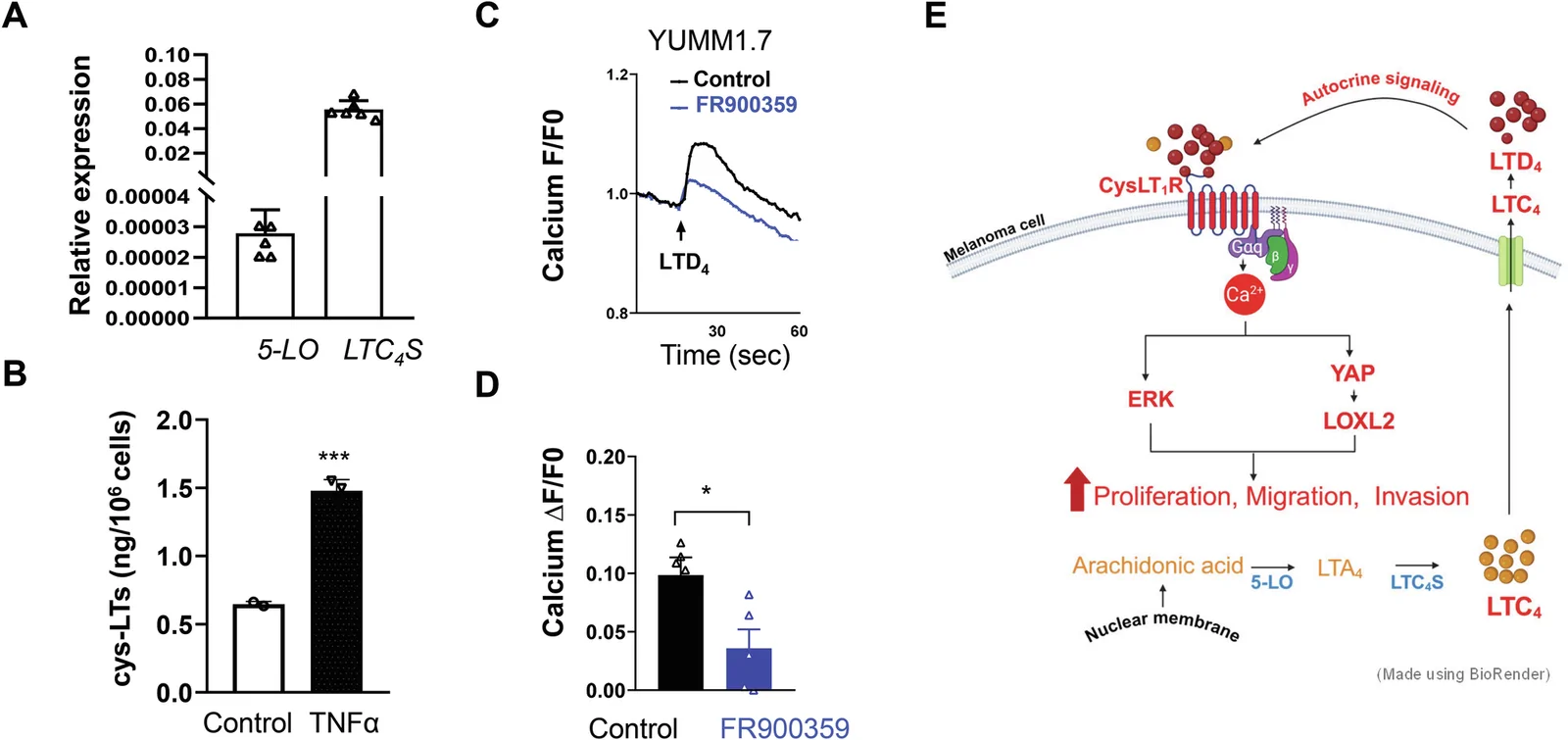
Melanoma cells foster CysLT_1_R-mediated autocrine signaling by secreting cys-LTs and CysLT_1_R flux calcium via Gαq subunit. **A** YUMM1.7 cells were assessed for 5-LO and LTC_4_S transcript expression through qPCR analysis. **B** Concentration of cys-LTs in the supernatant of YUMM1.7 was assayed by cys-LT ELISA in the presence or absence of TNF-α (10 ng/mL) treated for 12 h. **C** Representative traces displaying relative changes in cytosolic calcium in response to LTD_4_ (100 nM), in Fluo-4-loaded cells pre-treated with or without Gαq subunit antagonist, FR900359 (1 µM). Arrow denotes the time when the cells were stimulated with LTD_4_. **D** Quantitative analysis of cytosolic calcium influx calculated as ∆F/F0 which is the ratio of normalized Fluo-4 fluorescence intensity relative to time 0. **E** Cartoon depicting putative mechanisms of autocrine CysLT_1_R-mediated melanoma cell proliferation, migration, and invasion in vitro. All data representations are means ± SD from three independent experiments (*n* = 3). D’Agostino & Pearson test was performed to test if the sample distribution is normal, and significance was determined using ANOVA. Comparisons between the groups were determined by Tukey’s multiple comparisons test. *P < 0.05, ***P < 0.001.

Our previous results unraveled a surprising finding that knockdown of CysLT_1_R reduced basal expression of ECM components (Supplementary Fig. 2A–G) even in the absence of the ligand. Therefore, we wondered if melanoma cells, like other tumor cells could express cys-LT biosynthetic enzymes [29] to synthesize cys-LTs and trigger an autocrine loop. To test this, we first evaluated if melanoma cells expressed the two major enzymes required for cys-LT synthesis, 5-LO and LTC_4_S enzymes. Interestingly, qPCR analysis revealed appreciable mRNA levels of both 5-LO and LTC_4_S genes in YUMM1.7 cells (Fig. 6A). Further YUMM1.7, B16F10, and WM266-4 cells released detectable amount of cys-LTs into their culture media as detected by cys-LT ELISA (Fig. 6B and Supplementary Fig. 2H). Cys-LT release in YUMM1.7 is further augmented in the presence of pro-inflammatory mediator, tumor necrosis factor-alpha (TNF-α) (Fig. 6B).

### Cys-LT_1_R signals via Gαq-dependent signaling axis

We next asked how LTD_4_/CysLT_1_R axis relayed downstream signals. Since LTD_4_ efficiently fluxed calcium in all melanoma cell lines (Fig. 1C), we wondered if Gαq is activated downstream of CysLT_1_R. In agreement, a specific Gαq inhibitor FR900359 significantly attenuated LTD_4_-induced calcium in YUMM1.7 cells (Fig. 6C, D). Prior studies have established the crucial role of Gαq in activating ERK [30] and YAP [31] pathways in cancer leading to its progression. Taken together, our in vitro results reveal a unique autocrine LTD_4_/CysLT_1_R/Gαq/ERK-YAP signaling loop regulating melanoma cell proliferation and invasion (Fig. 6E).

### CysLT_1_R promotes melanoma growth in mice

**Fig. 7:**
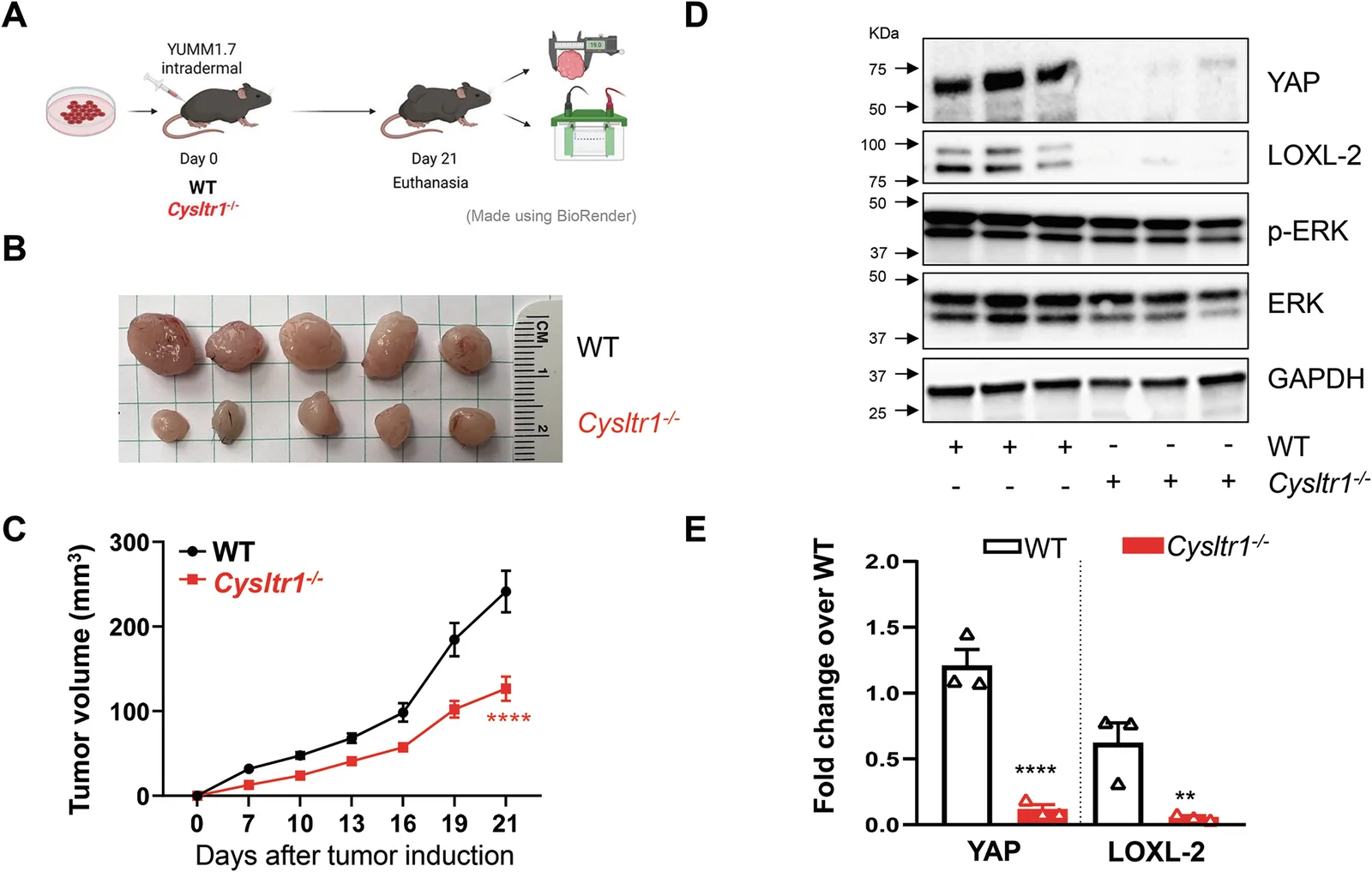
CysLT_1_R promotes melanoma progression in an orthotopic model. **A** Cartoon depicting orthotopic model of melanoma in mice. YUMM1.7 cells (0.1 × 10^6^ in 50 μL of HBSS) were injected intradermally into both flanks of the indicated mouse groups. On day 21, mice were euthanized, **B** tumors were harvested and **C** tumor volumes were measured. **D** Tumors from WT and *Cysltr1*^*–/–*^ mice were assessed for YAP, LOXL-2 and ERK protein expression by western blotting and **E** quantified. Data in **C** represents means ± SEM from a total of *n* = 21 tumors per group from three independent experiments, and **E** represents means ± SD from three independent experiments (*n* = 3). D’Agostino & Pearson test was performed to test if the sample distribution is normal, and significance was determined using ANOVA. Comparisons between the groups were determined by Tukey’s multiple comparisons test. **P < 0.01, ****P < 0.0001.

To determine if CysLT_1_R mediates melanoma growth in vivo, we implanted 1x10^6^ murine melanoma cells (YUMM1.7 and B16F10) subcutaneously under the flank of WT and *Cysltr1*^*–/–*^ C57/BL6 mice (ectopic model), and measured tumor growth over a period of 21 days (Supplementary Fig. 3A). In YUMM1.7-bearing mice and B16F10-bearing mice, tumors were significantly smaller in *Cysltr1*^*–/–*^ group compared to the WT group from day 14 and day 18 respectively through the end of the study (Supplementary Fig. 3B–E). Additionally, since melanoma emerges from the epidermal layer of the skin, we performed an orthotopic induction of melanoma by injecting 0.1x10^6^ YUMM1.7 cells on the flank of WT and *Cysltr1*^*–/–*^ mice intradermally to better mimic the natural tumor microenvironment and observed disease progression (Fig. 7A). We observed similar results in this orthotopic model where *Cysltr1*^*–/–*^ mice showed significantly smaller tumor volumes compared to WT mice (Fig. 7B, C), and attenuation in total YAP protein (Fig. 7D, E) strongly suggesting the crucial role played by CysLT_1_R/YAP axis in melanoma tumor growth. Additionally, we observed reduced LOXL-2 transcript (Supplementary Fig. 5A) and protein expression (Fig. 7D, E) in *Cysltr1*^*–/–*^ mice tumors compared to WT tumors. In addition to YAP and LOXL-2, another key ECM protein, fibronectin, was also significantly downregulated at the transcript level in *Cysltr1*^*–/–*^ tumors compared to WT tumors (Supplementary Fig. 5A). Notably, we did not observe similar changes in phospho- and total-ERK levels (Fig. 7D).

### Pharmacological inhibition of CysLT_1_R significantly attenuated melanoma growth in vivo

**Fig. 8:**
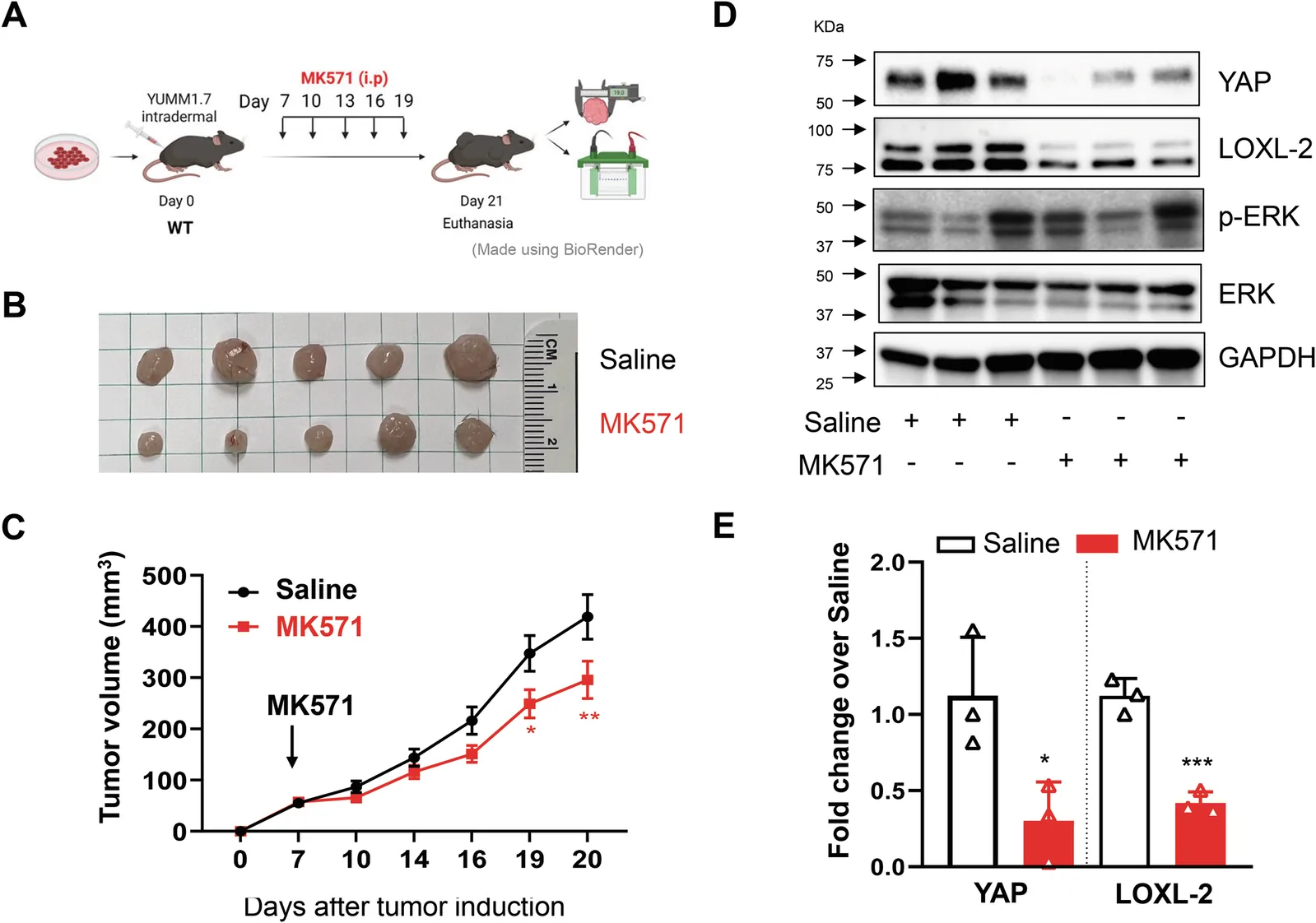
Pharmacological targeting of CysLT_1_R using MK571 inhibits melanoma progression. **A** Cartoon depicting orthotopic model of melanoma in mice followed by pharmacological targeting of CysLT_1_R with MK571. YUMM1.7 cells (0.1 × 10^6^ in 50 μL of HBSS) were injected intradermally into the flank of WT mice. When tumors were palpable on the seventh day (around 100mm^3^), WT mice were divided into 2 cohorts. First cohort of mice received 150 µL of saline by intraperitoneal (i.p) injection whereas the second cohort received MK571 (3 mg/kg of body weight in 150 µL of saline) by intraperitoneal (i.p) injection. Saline and MK571 were administered every three days till day 21, when mice were euthanized, **B** primary tumors were harvested, and **C** tumor volumes were measured. **D** Tumors from saline and MK571-treated mice were assessed for YAP, LOXL-2 and ERK protein expression by western blotting and **E** quantified. Data in **C** represents means ± SEM from a total of *n* = 24 tumors per group from three independent experiments, and **E** represents means ± SD from three independent experiments (*n* = 3). D’Agostino & Pearson test was performed to test if the sample distribution is normal, and significance was determined using ANOVA. Comparisons between the groups were determined by Tukey’s multiple comparisons test. *P < 0.05, **P < 0.01, ***P < 0.001.

Encouraged by the above findings, next we analyzed if pharmacological inhibition of CysLT_1_R could mimic *Cysltr1*^*–/–*^ phenotype, and if CysLT_1_R antagonist, MK571 could inhibit melanoma progression. WT mice were intradermally injected with YUMM1.7 cells (Fig. 8A) or subcutaneously injected with B16F10 cells (Supplementary Fig. 4A). When the tumor volumes reached around 100 mm^3^, saline or MK571 (3 mg/kg of body weight) were administered intraperitoneally every three days until day 19. MK571 treatment significantly attenuated YUMM1.7 (Fig. 8B, C) as well as B16F10 tumor volumes (Supplementary Fig. 4B, C) in comparison to saline treatment. Furthermore, pharmacological inhibition of CysLT_1_R with MK571 administration also successfully inhibited total YAP protein (Fig. 8D, E) and LOXL-2 expression at both mRNA (Supplementary Fig. 5B) and protein (Fig. 8D, E) levels. Fibronectin mRNA as seen in genetic model was found to be significantly downregulated in MK571-treated tumors compared to saline controls (Supplementary Fig. 5B). Notably, no significant changes in phospho- and total-ERK protein levels were observed between the two groups (Fig. 8D).

## Discussion

Our current study highlights CysLT_1_R as an oncogenic GPCR that drives melanoma growth and invasion through distinct ERK- and YAP/LOXL-2-dependent pathways. In parallel, we show that melanoma cells possess an autocrine cys-LT circuit that sustains CysLT_1_R/Gαq signaling. Genetic ablation or pharmacological inhibition of CysLT_1_R attenuates tumor growth and reduces YAP, LOXL-2, and fibronectin in subcutaneous and orthotopic models, nominating CysLT_1_R as a tractable therapeutic node in melanoma.

Chronic inflammation is a well-established driver of tumor initiation and can reprogram the tumor ECM to favor invasion and immune evasion [32]. Cysteinyl leukotrienes are potent inflammatory mediators that couple to CysLT_1_R/CysLT_2_R/ CysLT_3_R, which are primarily implicated in chronic asthma and airway hyper-responsiveness in humans and mice [33, 34]. Beyond airway biology, we and others have previously studied their role in promoting proliferative and migratory responses in multiple normal cell types [14–19] and cancers, including lung cancer [35], colon cancer [36], glioblastoma [37], and non-Hodgkin lymphomas [38]. In uveal melanoma, high CysLT_1_R expression associates with poor prognosis, and CysLT_1_R antagonists reduced cell proliferation and survival [12, 13]. Consistent with these observations, our analysis of uveal melanoma bulk RNA-seq datasets of patients available in the University of Alabama at Birmingham Cancer data analysis (UALCAN) portal revealed that higher CysLT_1_R transcript expression correlates with worse survival (not shown). However, the role of CysLT_1_R in cutaneous melanoma has remained largely correlative, limited to expression analyses and preliminary functional reports, with limited mechanistic interrogation.

Analysis of TCGA/bulk RNA-seq datasets revealed CysLT_1_R transcript upregulation in metastatic vs. primary cutaneous melanoma tumors, prompting functional interrogation in physiologically relevant models: murine B16F10 and YUMM1.7 (harboring *Braf*^V600E^/*Pten*^−/−^/*Cdkn2a*^−/−^ mutations recapitulating human disease) and human WM266-4 melanoma lines. All three cell lines expressed functional CysLT_1_R as evidenced by ligand-evoked calcium flux that was subsequently inhibited by a CysLT_1_R antagonist, MK571. While LTD_4_/CysLT_1_R signaling conferred a significant, albeit quantitatively moderate, proliferative advantage to melanoma cells cultured in vitro, the downstream pathways it mobilized were distinct and collectively supported tumor progression. Mechanistically, we define two major outputs of CysLT_1_R signaling in melanoma originating from Gαq activation. First, activation of the LTD_4_/CysLT_1_R axis induces ERK phosphorylation, and ERK inhibition or CysLT_1_R loss abrogates LTD_4_-driven proliferation, migration, and invasion. These findings align with prior work showing that CysLT_1_R-ERK coupling supports proliferation in mast cells, endothelial cells, and intestinal epithelial cells, and promotes migration [15, 17, 18, 20, 39]. Second, we uncovered a novel CysLT_1_R-mediated YAP-LOXL-2 axis that operates independently of ERK. CysLT_1_R knockdown reduces YAP/LOXL-2 expression and ECM remodeling signatures (fibronectin, vimentin, MMP-2, Twist, and Snail). YAP is a central mechanosensory effector that controls transcriptional programs for proliferation, migration, and invasion [40, 41]. Further, its effector LOXL-2 augments matrix stiffness through collagen cross-linking, promoting epithelial-mesenchymal transition (EMT), facilitating tumor cell invasion and migration [26, 27]. Although some studies place ERK as an upstream effector of YAP [42], our data position CysLT_1_R as a common upstream regulator of both ERK and YAP pathways independently. Our data joins CysLT_1_R to the emerging class of melanoma-relevant GPCRs and offers an indirect strategy to temper YAP activity where direct YAP targeting has proven challenging [40].

We further demonstrate that melanoma cells can self-sustain CysLT_1_R signaling via an autocrine cys-LT circuit. They express the biosynthetic enzymes 5-LO and LTC_4_S and release cys-LTs at baseline and in response to inflammatory cues. This autocrine circuit likely stabilizes a mesenchymal, motile state to facilitate melanoma progression and invasion by maintaining YAP/LOXL-2 programs. This aligns with observations that various cancers, including breast cancer cells, upregulate 5-LO [43–45]. Although attenuation of FN, YAP, and LOXL-2 expression following CysLT_1_R knockdown under basal conditions supports the existence of endogenous autocrine signaling independently of exogenous ligand addition, it is to be noted that higher exogenous LTD_4_ concentrations were used during in vitro stimulations, consistent with prior literature, to assess maximal receptor-dependent intracellular signaling. We presume that the measured cys-LT concentrations represent bulk accumulated extracellular levels and likely underestimate the effective local concentrations experienced at the cell surface during autocrine signaling. Further, cys-LTs are highly labile lipid mediators, and our media measurements may not accurately reflect biologically active microenvironmental concentrations. We speculate that endogenous cys-LTs function through chronic low-level autocrine signaling to sustain basal mesenchymal and migratory gene programs under unstimulated conditions. In contrast, higher exogenous LTD_4_ concentrations may be required to elicit acute signaling outputs such as proliferation and migration, which likely require higher receptor occupancy and/or stronger signal amplification. It is possible that distinct biological outputs could depend on different thresholds of CysLT_1_R signaling. Further, tumor cells in the TME could experience a higher concentration of cys-LTs released by other neighboring cells, promoting tumor growth.

In vivo, both genetic deletion of CysLT_1_R and systemic administration of CysLT_1_R antagonist (MK571) suppressed tumor growth and reduced YAP, LOXL-2, and FN in subcutaneous and orthotopic melanoma models, confirming the functional relevance of CysLT_1_R-YAP/LOXL-2 signaling in the native tumor microenvironment. Further, *Ltc4s*^*–/–*^ mice mirrored *Cysltr1*^*–/–*^ mice in our preliminary in vivo intradermal tumor experiments (data not shown), suggesting that both the absence of the ligands and the receptor have similar effects. Notably, tumor reduction in global *Cysltr1*^*–/–*^ mice despite receptor retention on melanoma cells suggests a non-redundant contribution from host (non-tumor) CysLT_1_R. Our findings suggest that CysLT_1_R signaling exerts both tumor cell-intrinsic and microenvironment-dependent functions during melanoma progression. In vitro, CysLT_1_R was required for melanoma cell proliferation and migration, supporting a direct role in melanoma cells. However, despite intact tumor-cell CysLT_1_R expression, tumor growth was markedly reduced in CysLT_1_R-deficient hosts, demonstrating that host CysLT_1_R signaling is a critical determinant of tumor progression in vivo. These findings are not mutually exclusive but instead highlight the context-dependent nature of CysLT_1_R signaling. While cell-autonomous effects are readily evident under isolated in vitro conditions, tumor progression in vivo additionally depends on complex stromal, vascular, and immune interactions within the tumor microenvironment [46, 47]. Furthermore, analysis of melanoma single-cell RNA-seq datasets extracted from TCGA [48] demonstrated relatively higher CysLT_1_R expression in B cells and macrophages (Supplementary Fig. 6), suggesting that these immune populations could serve as key mediators of CysLT_1_R-dependent signaling within the TME. Thus, loss of host CysLT_1_R signaling likely disrupts multiple complementary pro-tumorigenic signals that collectively outweigh the melanoma cell-intrinsic in vitro effects supporting CysLT_1_R role in melanoma progression via both cell-autonomous and non-cell-autonomous mechanisms, with a dominant role for microenvironmental contribution in vivo. Importantly, systemic inhibition of CysLT_1_R by MK571 recapitulated the knockout phenotype, suggesting that the pharmacologic efficacy likely stems from concurrent disruption of tumor intrinsic proliferation/invasion signals and CysLT_1_R-medited TME crosstalk. Our findings, therefore, have important therapeutic implications. CysLT_1_R is a druggable target, and the CysLT_1_R-specific antagonist Singulair (Montelukast) has been FDA-approved for asthma management for over three decades, supporting its potential repurposing for the treatment of melanoma with minimal side effects [49].

Collectively, our data provide the first comprehensive mechanistic dissection of CysLT_1_R as an oncogenic GPCR driving melanoma proliferation and invasion. CysLT_1_R blockade is a strategy to simultaneously blunt ERK and YAP/LOXL-2-driven melanoma cell proliferation and invasion while also disrupting CysLT_1_R-supported TME cues. Our strategy could complement existing MAPK-targeted therapy and immune checkpoint blockade in melanoma [50]. Future studies are warranted to determine the expression levels of CysLT_1_R protein in melanoma patients and delineate the specific immune cell populations within TME that rely on CysLT_1_R signaling to support melanoma progression. Defining whether CysLT_1_R expression or cys-LT biosynthetic signatures can effectively stratify melanoma patients who are most likely to benefit from CysLT_1_R-targeted therapies will also be essential. In parallel, investigating the mechanistic crosstalk between CysLT_1_R signaling and the current targeted or immunotherapies may uncover rational combinations capable of improving long-term clinical outcomes in aggressive melanoma.

## Supplementary information

Supplemental material text and figures merged

## Data Availability

The data to support the findings of the present study are available from the corresponding author upon request.
