# Supplementary material for: Autocrine CysLT1R-ERK/YAP signaling drives melanoma progression and reveals a targetable oncogenic GPCR axis
Source: Oncogene. 2026 Jul 24;45(35):3673–83. doi: 10.1038/s41388-026-03919-1 (PMC13503276; doi:10.1038/s41388-026-03919-1)
Supplement: Supplementary file 1 [file 41388_2026_3919_MOESM1_ESM.pdf]

Autocrine $CysLT_1R$-ERK/YAP signaling drives melanoma progression and reveals a targetable oncogenic GPCR axis

Emma Elizabeth Sabu Kattuman [1], Lakshminarayan Reddy Teegala [1], Venkatesh Katari [1], Somayeh Darzi [1], Srinivas Vinod Saladi [2], Ivana de le Serna [2], Charles K Thodeti [1], Sailaja Paruchuri [1*]

[1] Department of Physiology and Pharmacology, [2] Department of Cell and Cancer Biology, The University of Toledo College of Medicine and Life Sciences, Toledo, OH.

Running Title: $CysLT_1R$-ERK/YAP axis in melanoma progression

***Corresponding Author:**

Sailaja Paruchuri, PhD, Department of Physiology and Pharmacology, Block Health Science Building, 3000 Arlington Ave, University of Toledo, Toledo, OH 43614; Phone: 4193834465; E-mail: Sailaja.Paruchuri@UToledo.edu

Competing Interests:

The authors declare no competing interests.

Key words:

$CysLT_1R$, ERK, YAP, Gαq, tumor, proliferation, invasion

Supplementary Table 1

List of qPCR primers:

Gene name	Forward primer sequence	Reverse primer sequence
mouse Fibronectin	CCCTATCTCTGATACCGTTGTCC	TGCCGCAACTACTGTGATTCGG
mouse Vimentin	CGGAAAGTGGAATCCTTGCAGG	AGCAGTGAGGTCAGGCTTGGAA
mouse GAPDH	CTCCCACTCTTCCACCTTCG	CCACCACCCTGTTGCTGTAG
mouse $CysLT_1R$	CATTGCCTCTCCGTGTGGTCTA	TGGCTGTCATAAAGAAGATGCTAC
mouse MMP-2	CAAGGATGGACTCCTGGCACAT	TACTCGCCATCAGCGTTCCCAT
mouse 5-LO	TCTTCCTGGCACGACTTTGCTG	GCAGCCATTCAGGAACTGGTAG
mouse LTC_4S	CCTACAGGTGATCTCTGCACGA	TGGCGAGGAACAGCGGAAAGTA
mouse LOXL-2	TTCTGCCTGGAGGACACTGAGT	TCGGTGATGTCTATCCACTGGC
mouse YAP1	CCAGACGACTTCCTCAACAGTG	GCATCTCCTTCCAGTGTGCCAA
mouse Twist1	GATTCAGACCCTCAAACTGGCG	AGACGGAGAAGGCGTAGCTGAG
mouse Snail	TGTCTGCACGACCTGTGGAAAG	CTTCACATCCGAGTGGGTTTGG

Supplementary figure legends

Supp. Fig 1: LTD_4 promotes proliferation, migration, and invasion of human and mouse melanoma cells: (A) WM266-4 (human melanoma cell line) was treated with or without LTD_4 (100nM) for 72h followed by BrdU to assess their proliferation rate. **(B, D)** 10^4 cells (WM266-4 and B16F10) per well were seeded in a 96-well plate. When the cells are confluent, a scratch was made in the center of each well using the WoundMaker™ followed by stimulating the cells with LTD_4. Plate was then transferred to IncuCyte® S3 Live-Cell Analysis System to capture the closure of the scratch wound by the cells over a period of 24h. **(C)** $3x10^5$ WM266-4 cells in 200uL serum-free media were seeded on Matrigel® pre-coated trans-well inserts of 8.0 µm pore size placed in a 24-well plate. The bottom chamber comprised of 750uL of 10% FBS. Cells were stimulated with or without LTD_4 (100nM) and were allowed to invade for 24h after

which membranes were fixed and stained with crystal violet and quantified. All data representations are means ± SD from three independent experiments (n=3). D'Agostino & Pearson test was performed to test if the sample distribution is normal, and significance was determined using ANOVA. Comparisons between the groups were determined by Tukey's multiple comparisons test. *P < 0.05, ***P < 0.001.

Supp. Fig 2: $CysLT_1R$ knockdown downregulated key ECM genes and proteins: (A) $CysLT_1R$ was knocked down in YUMM1.7 cells using $CysLT_1R$siRNA as previously described. Expression levels of key ECM proteins such as **(A, C)** FN transcript and protein, **(B, C)** vimentin transcript and protein, **(E)** Twist1 transcript, **(F)** Snail transcript, and **(G)** MMP-2 transcript were determined using qPCR and western blotting. **(D)** is the quantification of (C). **(H)** cys-LT levels secreted by B16F10 and WM266-4 respectively were measured using the cys-LT ELISA. All data representations are means ± SD from three independent experiments (n=3). D'Agostino & Pearson test was performed to test if the sample distribution is normal, and significance was determined using ANOVA. Comparisons between the groups were determined by Tukey's multiple comparisons test. *P < 0.05, **P < 0.01.

Supp. Fig 3: Genetic deletion of $CysLT_1R$ inhibits melanoma tumor growth in mice: (A) Cartoon depicting ectopic melanoma model in mice. YUMM1.7 and B16F10 cells (1×10^6 in 100 µL of HBSS) were injected subcutaneously into the flanks of the indicated mouse groups. On day 21, mice were euthanized, **(B, D)** tumors were harvested and **(C, E)** tumor volumes were measured. Data in (C) represents means ± SEM from a minimum of n=16 tumors per group from a total of three independent experiments, and (E) represents means ± SEM from a minimum of n=20 tumors per group from a total of three independent experiments. D'Agostino & Pearson test was performed to test if the sample distribution is normal, and significance was determined using ANOVA. Comparisons between the groups were determined by Tukey's multiple comparisons test. *P < 0.05, **P < 0.01, ****P < 0.0001.

Supp. Fig 4: Pharmacological targeting of $CysLT_1R$ reduces tumor burden in an ectopic model of B16F10 tumor-bearing mice: (A) Cartoon depicting ectopic model of melanoma induction in mice followed by pharmacological targeting of $CysLT_1R$ with MK571. B16F10 cells (1×10^6 in 100 µL of HBSS) were injected subcutaneously into the flanks of WT mice. When tumors were palpable on the tenth day

(around 100mm^3), WT mice were divided into 2 cohorts. First cohort of mice received 150uL of saline and the second cohort received MK 571 (3 mg/kg of body weight in 150 µL of saline) by intraperitoneal (i.p) injections. Saline and MK571 were administered every three days till day 21, when mice were euthanized, **(B)** primary tumors were harvested, and **(C)** tumor volumes were measured. Data in (C) represents means ± SEM from a minimum of n=13 tumors per group from a total of three independent experiments. D'Agostino & Pearson test was performed to test if the sample distribution is normal, and significance was determined using ANOVA. Comparisons between the groups were determined by Tukey's multiple comparisons test. *P < 0.05.

Supp. Fig 5: Genetic and pharmacological targeting of CysLT$_1$R caused significant downregulation of YAP-associated ECM genes: Tumors from WT and *Cysltr1$^{-/-}$* mice were assessed for **(A)** LOXL-2, **(B)** FN transcripts using qPCR analysis. Tumors from saline and MK571-treated mice were assessed for **(C)** LOXL-2 and **(D)** FN transcripts using qPCR analysis. All data representations are means ± SD from three independent experiments (n=3). D'Agostino & Pearson test was performed to test if the sample distribution is normal, and significance was determined using ANOVA. Comparisons between the groups were determined by Tukey's multiple comparisons test. *P < 0.05, **P < 0.01.

Supp. Fig 6: Analysis of melanoma tumor datasets reveal transcriptional upregulation of CysLT$_1$R in B cells and macrophages: **(A)** UMAP visualization of single-cell RNA-seq data showing expression of CYSLTR1 (left) and annotated cell types (right). Cells are colored by log-normalized gene expression or cell identity, including T cells, B cells, NK cells, macrophages, endothelial cells, CAFs, and unidentified cells. **(B)** Dot plot showing CysLT$_1$R expression across annotated cell populations. Dot size represents the percentage of cells expressing the gene, and color intensity indicates scaled mean expression within each cell type.

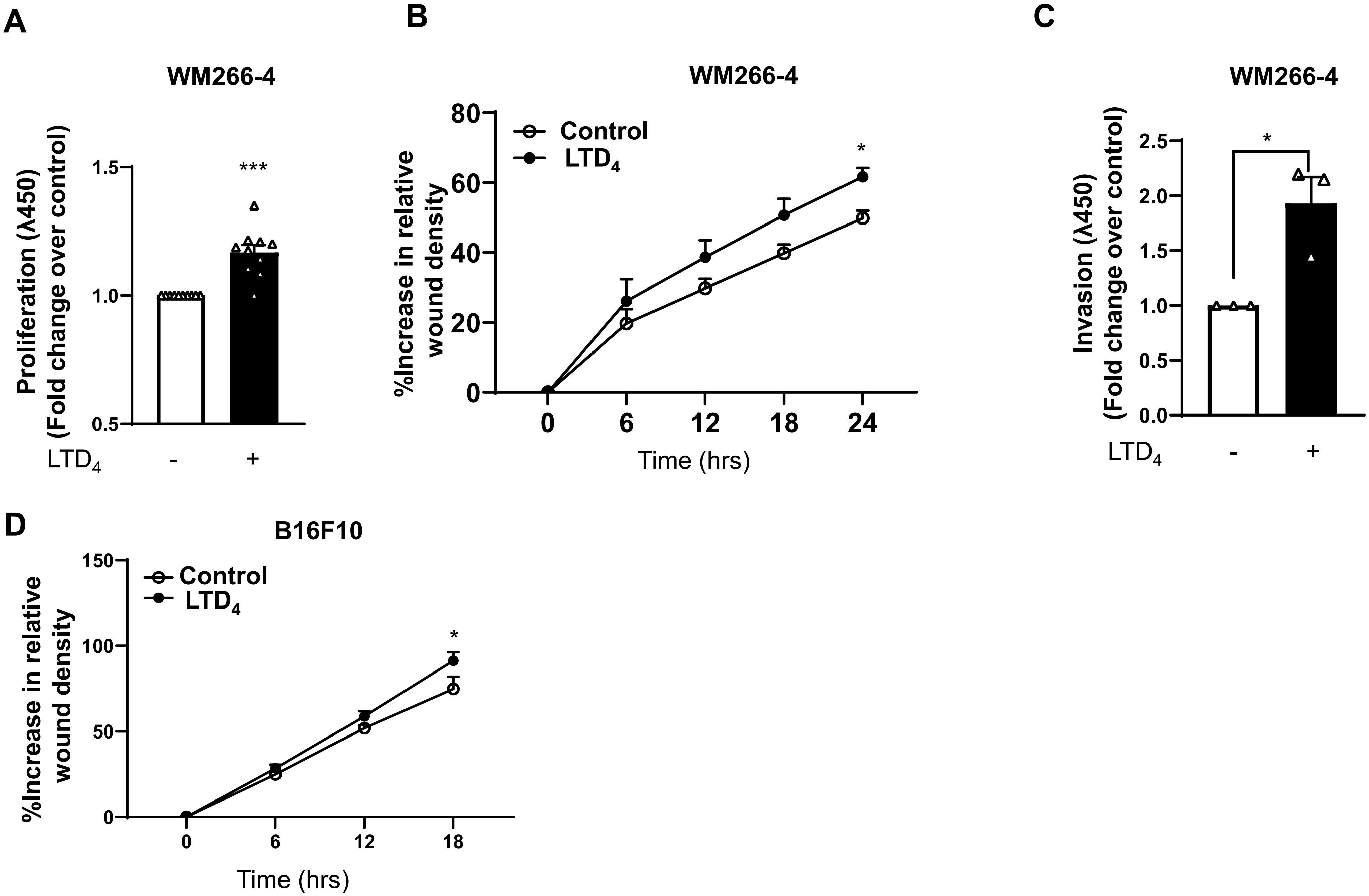
A
WM266-4
Proliferation (λ450)
(Fold change over control)
1.5
1.0
0.5
***
LTD4
-
+
B
WM266-4
80
60
40
20
0
Control
LTD4
*
%Increase in relative
wound density
0
6
12
18
24
Time (hrs)
C
WM266-4
Invasion (λ450)
(Fold change over control)
2.5
2.0
1.5
1.0
0.5
0.0
*
LTD4
-
+
D
B16F10
150
100
50
0
Control
LTD4
*
%Increase in relative
wound density
0
6
12
18
Time (hrs)

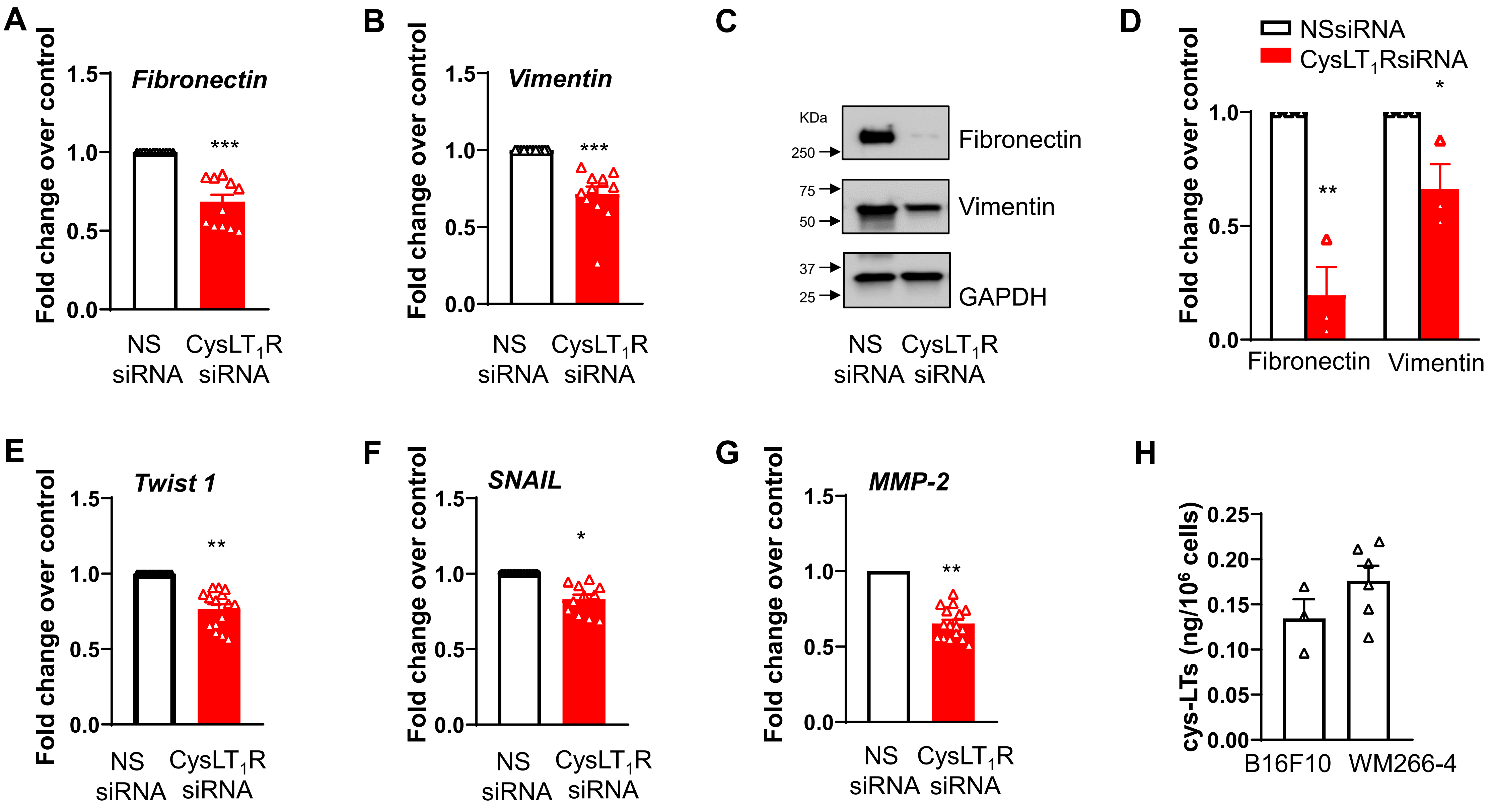

A
Fibronectin
Fold change over control
1.5
1.0
0.5
0.0
***
NS siRNA
CysLT1R siRNA
B
Vimentin
Fold change over control
***
NS siRNA
CysLT1R siRNA
C
KDa
250
75
50
37
25
Fibronectin
Vimentin
GAPDH
NS siRNA
CysLT1R siRNA
D
NSsiRNA
CysLT1RsiRNA
Fold change over control
**
*
Fibronectin
Vimentin
E
Twist 1
Fold change over control
**
NS siRNA
CysLT1R siRNA
F
SNAIL
Fold change over control
*
NS siRNA
CysLT1R siRNA
G
MMP-2
Fold change over control
**
NS siRNA
CysLT1R siRNA
H
cys-LTs (ng/10^6 cells)
0.25
0.20
0.15
0.10
0.05
0.00
B16F10
WM266-4

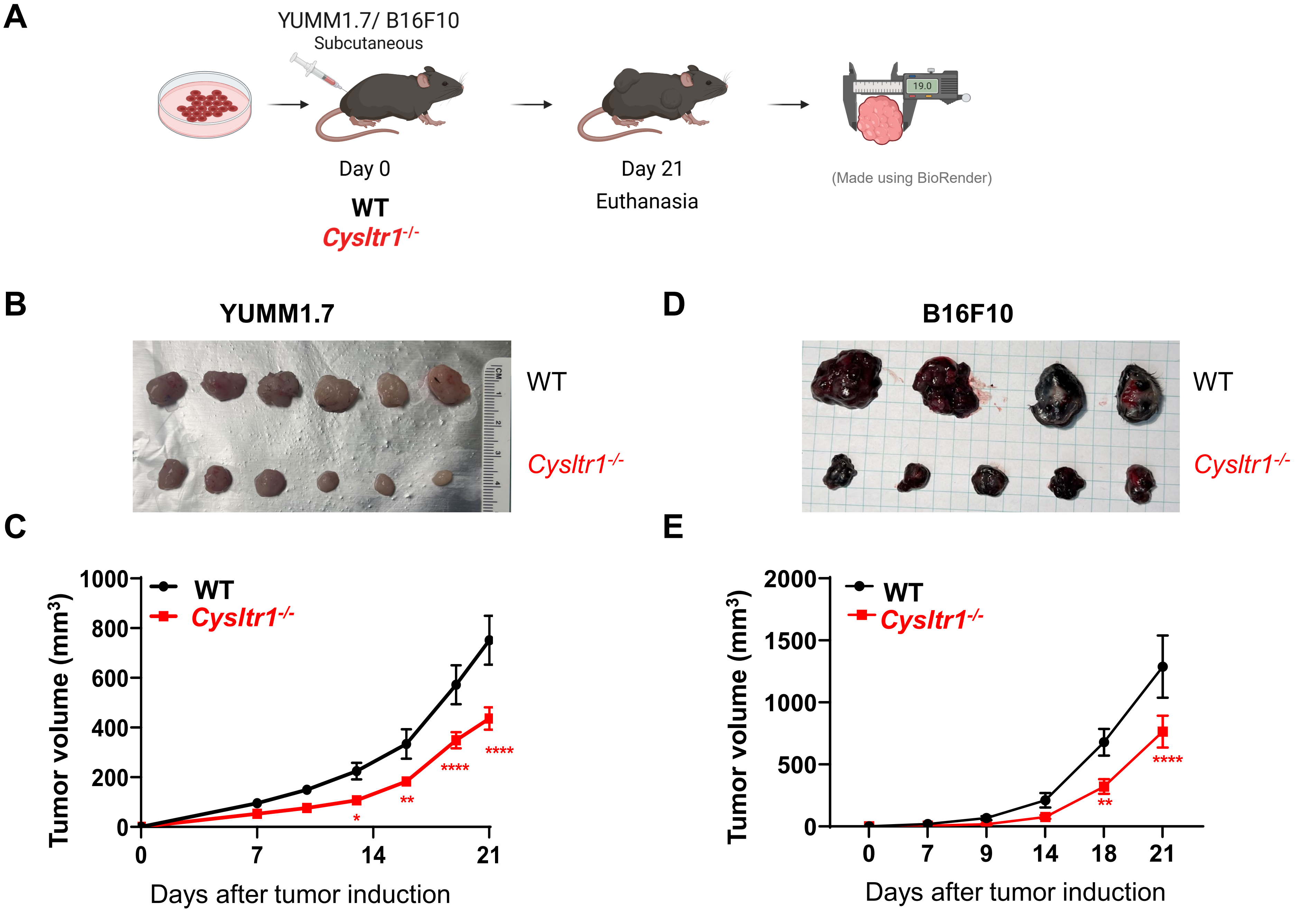
A
YUMM1.7/ B16F10
Subcutaneous
19.0
Day 0
WT
Cysltr1-/-
Day 21
Euthanasia
(Made using BioRender)
B
YUMM1.7
WT
Cysltr1-/-
D
B16F10
WT
Cysltr1-/-
C
Tumor volume (mm3)
1000
800
600
400
200
0
WT
Cysltr1-/-
0
7
14
21
Days after tumor induction
*
**
****
****
E
Tumor volume (mm3)
2000
1500
1000
500
0
WT
Cysltr1-/-
0
7
9
14
18
21
Days after tumor induction
**
****

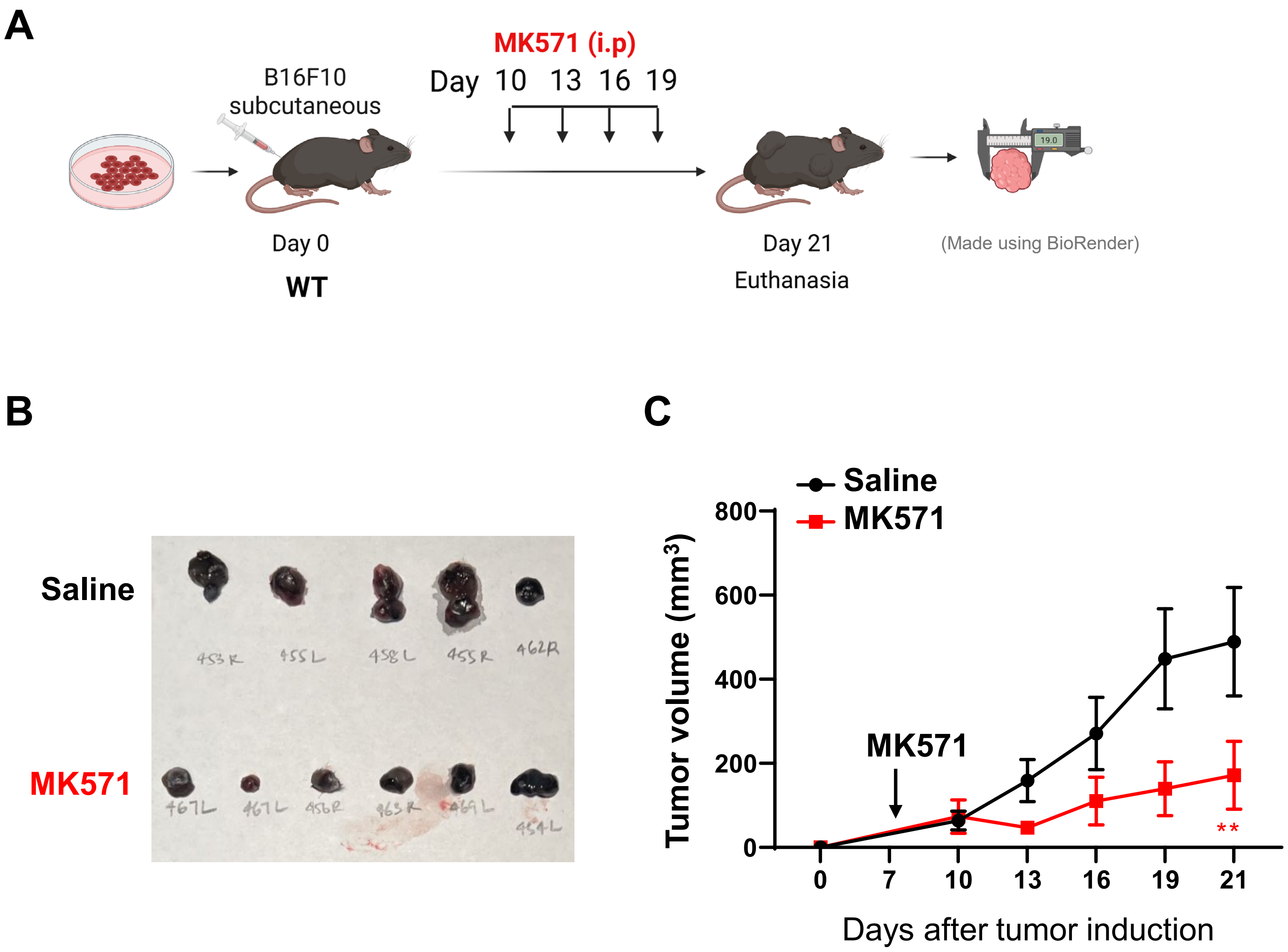
A
MK571 (i.p)
Day 10 13 16 19
B16F10
subcutaneous
Day 0
WT
Day 21
Euthanasia
(Made using BioRender)
B
Saline
MK571
C
Saline
MK571
Tumor volume (mm³)
800
600
400
200
0
MK571
**
0
7
10
13
16
19
21
Days after tumor induction

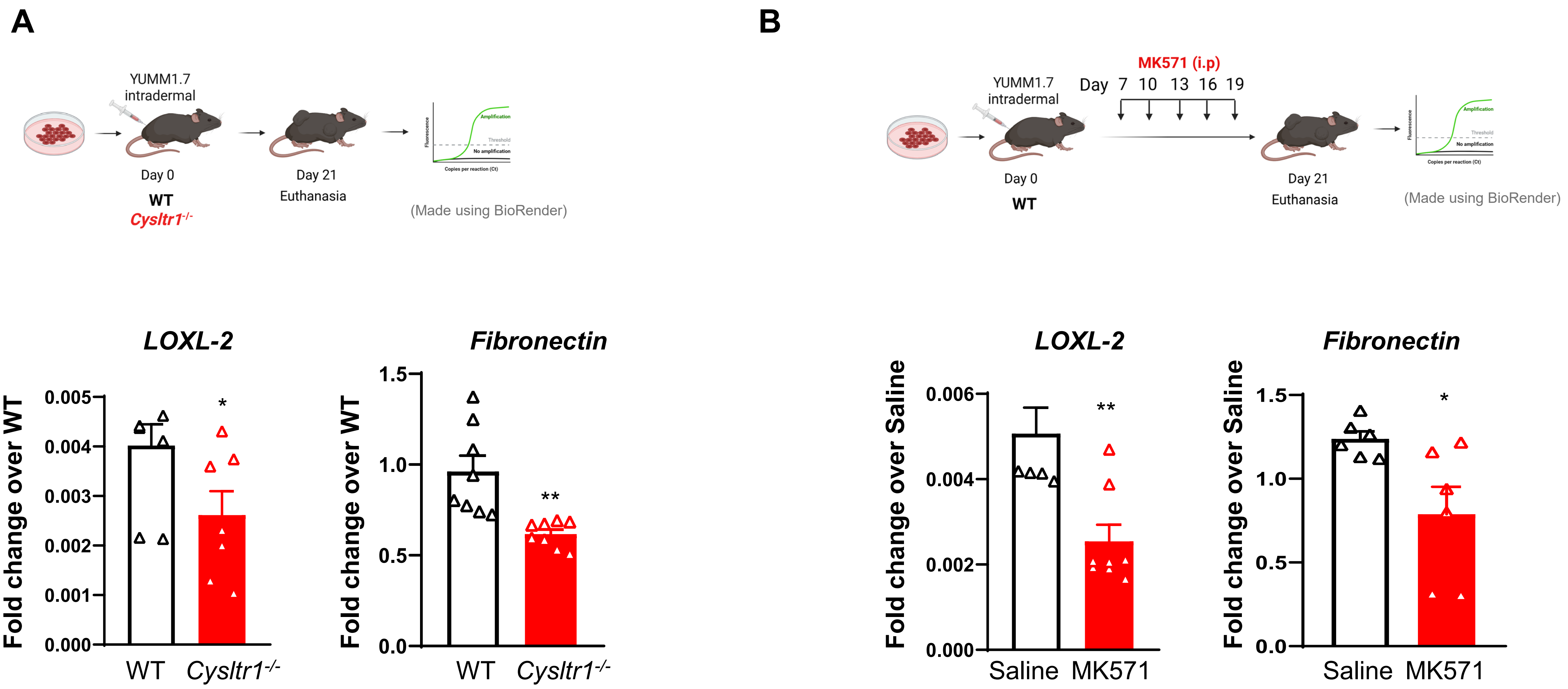

A
YUMM1.7 intradermal
Day 0
WT
Cysltr1-/-
Day 21
Euthanasia
(Made using BioRender)
LOXL-2
Fold change over WT
WT
Cysltr1-/-
Fibronectin
Fold change over WT
WT
Cysltr1-/-
B
MK571 (i.p)
Day 7 10 13 16 19
YUMM1.7 intradermal
Day 0
WT
Day 21
Euthanasia
(Made using BioRender)
LOXL-2
Fold change over Saline
Saline
MK571
Fibronectin
Fold change over Saline
Saline
MK571

A

B

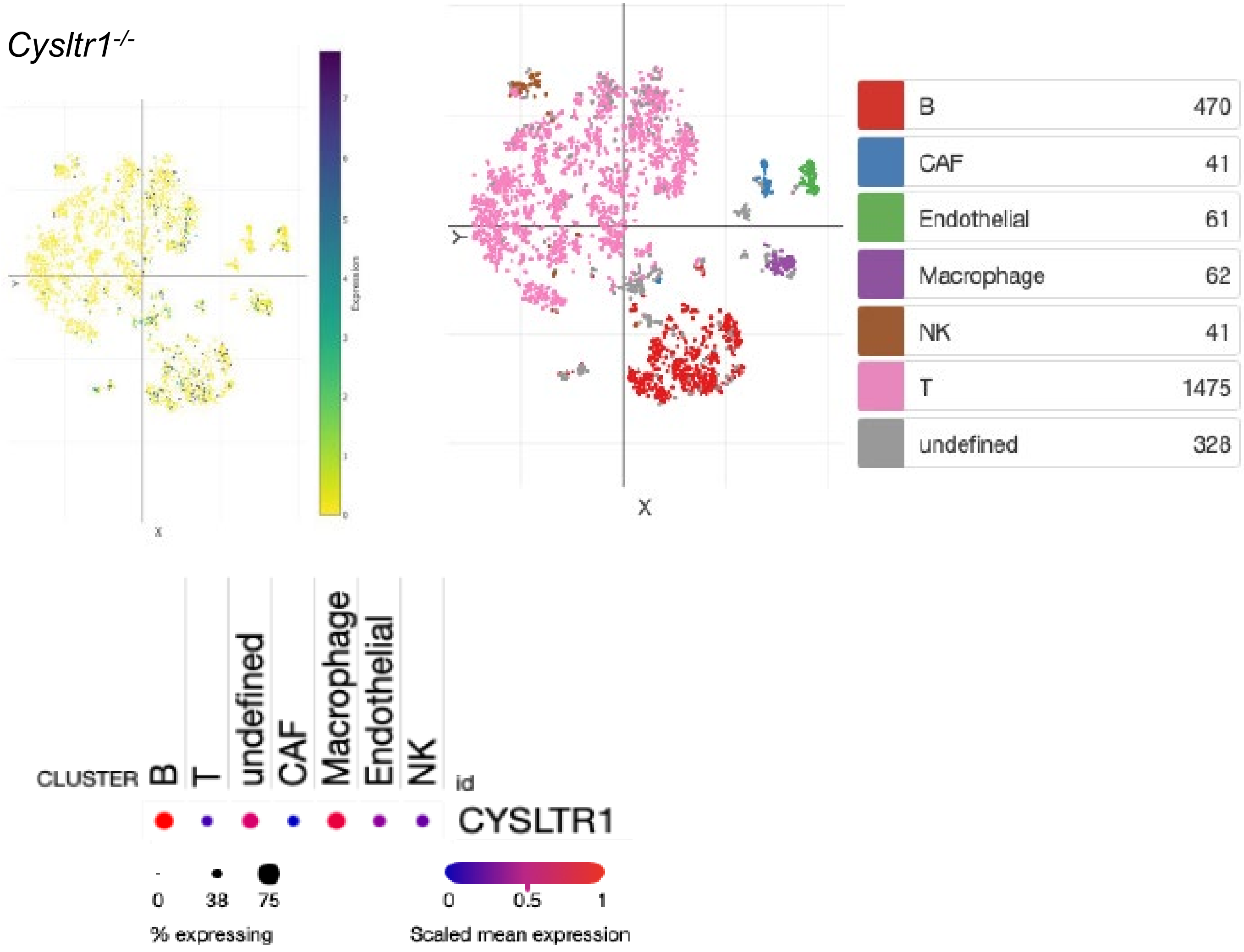
Cysltr1-/-
Expression
X
Y
B 470
CAF 41
Endothelial 61
Macrophage 62
NK 41
T 1475
undefined 328
CLUSTER
B
T
undefined
CAF
Macrophage
Endothelial
NK
id
CYSLTR1
0
38
75
% expressing
0
0.5
1
Scaled mean expression
